# The experimental hut efficacy of next-generation insecticide-treated nets against pyrethroid-resistant malaria vectors after 12, 24 and 36 months of household use in Benin

**DOI:** 10.1186/s12936-024-05199-0

**Published:** 2024-12-18

**Authors:** Abel Agbevo, Thomas Syme, Josias Fagbohoun, Augustin Fongnikin, Juniace Ahoga, Manfred Accrombessi, Natacha Protopopoff, Jackie Cook, Thomas S. Churcher, Gil G. Padonou, Arthur Sovi, Idelphonse Ahogni, Renaud Govoetchan, Damien Todjinou, Martin Akogbeto, Corine Ngufor

**Affiliations:** 1 London School of Hygiene and Tropical Medicine (LSHTM), London, WC1E 7HT UK; 2 Centre de Recherches Entomologiques de Cotonou (CREC), Cotonou, Benin; 3 Pan-African Malaria Vector Research Consortium (PAMVERC), Cotonou, Benin; 4 MRC Centre for Global Infectious Disease Analysis, Imperial College London, Norfolk Place, London, W2 1PG UK

**Keywords:** Insecticide-treated nets, Malaria, Vector control, Pyrethroid resistance, *Anopheles gambiae*

## Abstract

**Background:**

Cluster-randomized controlled trials (cluster-RCTs) have demonstrated variation in the epidemiological efficacy of different next-generation insecticide-treated net (ITN) types, with some providing shorter-lived impact than others. Further studies are needed to assess changes in the insecticidal durability of these ITNs over time to complement cluster-RCT results.

**Methods:**

A series of experimental hut trials were performed to evaluate the bioefficacy of new and field-aged next-generation ITNs (PermaNet^®^ 3.0, Royal Guard^®^, Interceptor^®^ G2) compared to a pyrethroid-only net (Interceptor^®^) against pyrethroid-resistant malaria vectors in Covè, southern Benin. Field-aged nets were withdrawn from households at 12, 24 and 36 months. Net pieces cut from whole ITNs were analysed for chemical content, and susceptibility bioassays were performed during each trial to assess changes in insecticide resistance in the Covè vector population.

**Results:**

Interceptor^®^ G2 induced superior mosquito mortality than the other ITNs across all time points. The improved mortality with Interceptor^®^ G2 compared to Interceptor^®^ was evident across all time points but was greater with new nets (odds ratio (OR) = 8.6, 95% CI [7.4, 10.1]) than field-aged nets (OR = 2.5, 95% CI [1.8, 3.5] at 12 months, OR = 2.4, 95% CI [1.6, 3.7] at 24 months and OR = 2.9, 95% CI [1.6, 5.1] at 36 months). New Royal Guard^®^ reduced mosquito fertility compared to the other ITNs, but this improvement fell after field-ageing, particularly at 24 months when it was similar to Interceptor^®^ (11% vs 3%, p = 0.08). When new, mortality was significantly higher with PermaNet^®^ 3.0 compared to Interceptor^®^ (OR = 3.6, 95% CI [3.0, 4.2]); however, this benefit was lost with field-aged nets at 12 months (OR = 1.1, 95% CI [0.8, 1.5]) and 24 months (OR = 0.6, 95% CI [0.4, 0.9]). Retention of the non-pyrethroid compound in next-generation nets was low after 36 months (27% for PermaNet^®^ 3.0, 26% for Royal Guard^®^ and 15% for Interceptor^®^ G2).

**Conclusions:**

Interceptor^®^ G2 outperformed the other ITNs, confirming the superiority of pyrethroid-chlorfenapyr nets over other net types. When new, all next-generation ITNs showed superior bioefficacy compared to Interceptor^®^; however, the size of this improvement fell after field-ageing due to poor durability of the non-pyrethroid compound. These findings emphasize the need to enhance the insecticidal durability of next-generation ITNs.

**Supplementary Information:**

The online version contains supplementary material available at 10.1186/s12936-024-05199-0.

## Background

Insecticide-treated nets (ITNs) have played a crucial role in reducing malaria morbidity and mortality over the past two decades. More than 3 billion ITNs have been distributed worldwide since 2004 [1], and modelling studies estimate that this rollout was responsible for 68% of the malaria cases averted in sub-Saharan Africa between 2000 and 2015 [2]. Unfortunately, resistance to pyrethroids—the standard insecticide class used on ITNs—has increased significantly in malaria vector populations [3], threatening to undermine the effectiveness of this core intervention. This has coincided with a stagnation in malaria control progress on a global scale and increases in case incidence in several high-burden countries since 2015 [4]. These trends have stimulated the development of a new generation of innovative ITN products designed to respond to the threat of pyrethroid resistance and help drive down stalling burden reductions towards ambitious global targets [5].

Currently, three types of next-generation ITN product are on the market, each combining a pyrethroid with another insecticide or synergist capable of overcoming pyrethroid resistance in malaria vectors. A series of cluster-randomized controlled trials (RCTs) have been performed in recent years to evaluate the epidemiological efficacy of these nets compared to standard pyrethroid-only nets and generate the evidence necessary to inform World Health Organization (WHO) recommendations. Nets combining a pyrethroid with piperonyl butoxide (PBO)—a synergist which enhances the potency of the pyrethroid by inhibiting mosquito detoxification enzymes [6]—were the first to receive WHO recommendation after two products (Olyset^®^ Plus and PermaNet^®^ 3.0) demonstrated superior epidemiological impact over pyrethroid-only nets in cluster-RCTs in Tanzania [7] and Uganda [8]. Further evidence is needed, however, to establish the public health value of pyrethroid-PBO nets in West Africa, where intense levels of pyrethroid resistance may diminish their benefit over pyrethroid-only nets [9].

More recently, dual-active ingredient (AI) ITN products combining a pyrethroid with either the pyrrole insecticide chlorfenapyr (CFP) (Interceptor^®^ G2) or the insect growth regulator pyriproxyfen (PPF) (Royal Guard^®^) have undergone parallel cluster-RCTs in Benin and Tanzania. The results of these trials provided strong evidence for the public health value of pyrethroid-CFP nets, as Interceptor^®^ G2 was shown to reduce malaria incidence by 46% and 44% over 2 years compared to pyrethroid-only nets in Benin [10] and Tanzania [11], respectively. In contrast, Royal Guard^®^ was shown to confer similar protection to pyrethroid-only nets in Benin [10] and Tanzania [11] and in a previous cluster-RCT in Burkina Faso, a discontinued pyrethroid-PPF net product (Olyset^®^ Duo) provided a modest 12% reduction in malaria incidence [12]. Based on this, the WHO has issued a strong recommendation for the deployment of pyrethroid-CFP nets and a conditional recommendation for pyrethroid-PPF nets over pyrethroid-only nets [13].

Coverage of next-generation ITNs is now being scaled up across malaria-endemic countries, renewing optimism that pyrethroid resistance can be tackled to reduce malaria cases and mortality further. But, there remain other threats to the effectiveness of ITNs beyond pyrethroid resistance, most notably their poor durability. To be considered long-lasting, ITNs must demonstrate physical and insecticidal durability for at least 3 years under operational conditions. However, an increasing body of evidence suggests that new nets fall below this threshold. Indeed, durability monitoring studies of pyrethroid-PBO nets in Kenya [14] and Uganda [15] revealed dwindling PBO content and failure to achieve WHO bioefficacy thresholds within 2 years of distribution. More recently, in cluster-RCTs, the superior impact of pyrethroid-PBO nets over pyrethroid-only nets lasted only 1 year in Tanzania [11], while the benefit of pyrethroid-CFP nets was lost in the 3rd year in Benin [16]. Studies evaluating the durability of next-generation ITNs and how this affects epidemiological performance will be crucial to informing the timing of distribution cycles, maintaining effective coverage and maximizing the impact of these nets as they are scaled up for full operational use.

Insecticidal durability of ITNs is typically evaluated by testing nets withdrawn from communities at regular intervals post-distribution in laboratory bioassays with insectary-reared mosquito strains [17]. While these assays are helpful in characterizing the bioavailability and potency of AI on the net surface over time, they provide limited information on the comparative performance of different types of next-generation net given that the methods and mosquito strains used must be adapted to the mode of action of the constituent AIs [18]. Experimental hut trials are an alternative method that can be used to evaluate the comparative performance of ITNs in durability studies under conditions that simulate the natural interaction between wild host-seeking mosquitoes and nets inside households during operational use. Indeed, recent modelling studies investigating entomological surrogates for epidemiological data have shown that the levels of mosquito mortality and blood-feeding measured in such trials can be used to predict the impact of ITNs on malaria incidence and prevalence in cluster-RCTs [19, 20]. Embedding experimental hut trials with field-collected ITNs within cluster-RCTs could, therefore, provide valuable data regarding the comparative bioefficacy and insecticidal durability of ITNs over their intended useful life and help explain the epidemiological results from these studies.

In this study, a series of experimental hut trials were performed to evaluate the efficacy of new and field-aged next-generation ITNs against a pyrethroid-resistant malaria vector population in Covè, southern Benin. Field-aged nets were withdrawn from communities near the experimental hut site after 12, 24 and 36 months of operational use by householders as part of a larger study to assess the durability of dual-active ingredient nets evaluated in the cluster-RCT in Benin [21, 22]. Next-generation ITN products from the pyrethroid-PBO (PermaNet^®^ 3.0), pyrethroid-PPF (Royal Guard^®^) and pyrethroid-CFP (Interceptor^®^ G2) net classes were compared to a standard pyrethroid-only net (Interceptor^®^). Net pieces cut from whole ITNs used in the experimental hut trials were sent for chemical analysis of AI content. WHO tube tests and bottle bioassays were also performed during each trial to assess longitudinal changes in the susceptibility of the local vector population to the AIs in the study ITNs and support interpretation of the experimental hut results.

## Methods

### Experimental hut trials

Experimental hut trials are standardized simulations of human-occupied housing recommended by the WHO for evaluating the efficacy of ITNs. These real-world assays recreate exposure conditions when host-seeking mosquitoes interact with nets inside households and are thus a highly suitable method for assessing the bioefficacy of operationally-aged nets throughout their intended lifespan.

### Study site and experimental huts

**Fig. 1:**
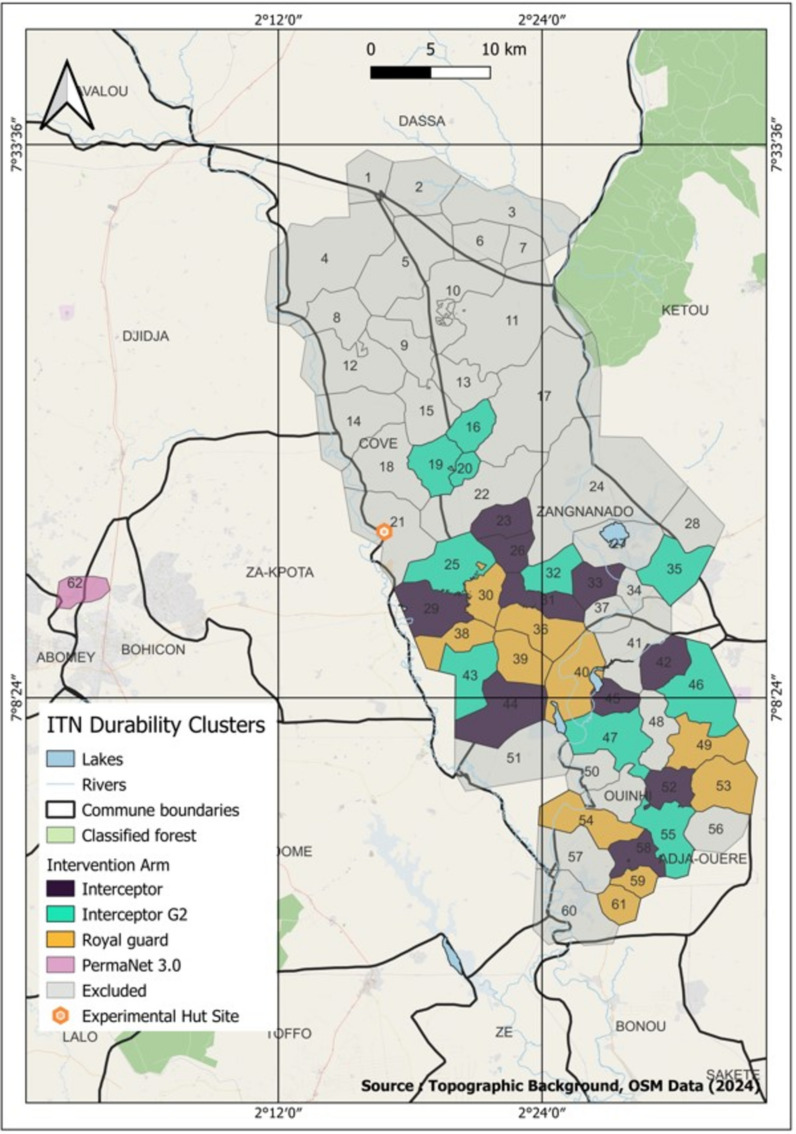
Map showing village clusters in Zou Department, Benin, where nets were withdrawn for insecticidal durability assessment. Interceptor^®^, Royal Guard^®^ and Interceptor^®^ G2 were distributed in the communes of Covè, Zagnanado and Ouinhi as part of a cluster-randomized controlled trial evaluating the epidemiological efficacy of next-generation nets. PermaNet^®^ 3.0 nets were distributed in the communes of Djidja and Bohicon during a mass campaign conducted by the national malaria control programme

The experimental hut trials were conducted in the commune of Covè, Zou Department, southern Benin (7° 14′ N2° 18′ E) near villages where field-aged study nets were withdrawn (Fig. 1). The huts are located in a large area of rice irrigation, which provides permanent and extensive breeding sites for mosquitoes and, hence, a high year-round density of malaria vectors. The vector population consists of a mixture of *Anopheles coluzzii* and *Anopheles gambiae *sensu stricto, with the latter occurring at lower proportions (~ 23%) and predominantly in the dry season [23]. Previous studies characterizing the resistance profile of the vector population using WHO susceptibility bioassays have demonstrated a high frequency (> 90% bioassay survival) and intensity (200-fold) of pyrethroid resistance but continued susceptibility to other insecticides including CFP and PPF [23–25]. Pre-exposure to PBO provides partial to complete restoration in pyrethroid susceptibility, demonstrating the contribution of P450s to observed pyrethroid resistance [24]. Genotyping and gene expression studies support this, revealing a high frequency of the knockdown resistance (*kdr*) mutation (89%) and four-fold overexpression of CYP6P3—a P450 validated as an efficient metabolizer of pyrethroids [23]. The experimental huts used were of standard West African design, made of concrete bricks with cement-plastered walls enclosed by a corrugated iron roof and a polyethylene ceiling. Each hut had a wooden-framed veranda projecting from the rear wall to capture exiting mosquitoes and was surrounded by a water-filled moat to prevent mosquito predators from entering.

### Description of study nets

In this study, the entomological efficacy of three different types of field-aged next-generation ITN (PermaNet^®^ 3.0, Royal Guard^®^ and Interceptor^®^ G2) was evaluated in experimental huts compared to a standard pyrethroid-only net (Interceptor^®^). All of these ITN products are on the WHO list of prequalified vector control products [26]. A detailed description of the specifications of each net is provided below:

i. Interceptor^®^ is a standard pyrethroid-only net manufactured by BASF. It is made of 100-denier polyester filaments coated with 5 g/kg (200 mg/m^2^) ± 25% of alpha-cypermethrin. Its dimensions are 1.8 (L) × 1.8 (W) × 1.8 (H) m, while the fabric weighs 40 g/m^2^ and has a minimum bursting strength of 405 kPa.
ii. PermaNet^®^ 3.0 is a pyrethroid-PBO net manufactured by Vestergaard Sàrl. It features a mosaic design consisting of 100-denier polyester side panels coated with 2.1 g/kg (84 mg/m^2^) ± 25% of deltamethrin and a 100-denier polyethylene roof panel incorporated with a mixture of deltamethrin and PBO at 4 g/kg (120 mg/m^2^) ± 25% and 25 g/kg (800 mg/m^2^) ± 25%, respectively. Its dimensions are 1.6 (L) × 1.8 (W) × 1.5 (H) m. The fabric weight and minimum bursting strength are 40 g/m^2^ and 350 kPa on the sides compared to 30 g/m^2^ and 400 kPa on the roof.
iii. Royal Guard^®^ is a pyrethroid-PPF net manufactured by Disease Control Technologies. It is made of 150-denier high-density polyethylene filaments incorporated with a mixture of alpha-cypermethrin and PPF at target doses of 5.0 g/kg (225 mg/m^2^) ± 25% each. Its dimensions are 1.8 (L) × 1.8 (W) × 1.6 (H) m while the fabric weighs 45 g/m^2^ and has a minimum bursting strength of 450 kPa.
iv. Interceptor^®^ G2 is a pyrethroid-CFP net manufactured by BASF. It is made of 100-denier polyester filaments coated with a mixture of alpha-cypermethrin and CFP at target doses of 2.4 g/kg (100 mg/m^2^) ± 25% and 4.8 g/kg (200 mg/m^2^) ± 25%, respectively. Its dimensions are 1.8 (L) × 1.8 (W) × 1.8 (H) m while the fabric weighs 40 g/m^2^ and has a minimum bursting strength of 405 kPa.

### Withdrawal of field-aged nets

The field-aged nets used for the experimental hut trials were distributed during a mass campaign in March 2020 to villages near the hut station in Zou Department, southern Benin. The Interceptor^®^, Royal Guard^®^ and Interceptor^®^ G2 nets were withdrawn from randomly selected clusters in the communes of Covè, Zagnanado and Ouinhi as part of a durability monitoring study nested within a cluster-RCT evaluating the epidemiological efficacy of dual-AI ITNs [21]. The PermaNet^®^ 3.0 nets were collected from the nearby village of Avokanzoun in the communes of Djidja and Bohicon (7° 20ʹ N 1° 56ʹ E), where they were distributed at the same time as the cluster-RCT nets during the 2020 mass campaign conducted by the national malaria control programme. The location of the study clusters/villages where the different ITN types were collected relative to the experimental hut site is shown in Fig. 1.

The design of the durability study, including the sampling of nets for the experimental hut trials, has been described previously [21]. To summarize, two cohorts of nets were marked at baseline from 10 randomly selected clusters of each arm of the cluster-RCT (Interceptor^®^, Royal Guard^®^ and Interceptor^®^ G2) and followed every 6–12 months to assess attrition and fabric integrity (cohort 1) as well as bioefficacy and chemical content (cohort 2) according to WHO guidelines [17]. Cohort 2 consisted of 1800 nets from 600 households per study arm that were collected (and replaced with new nets) and tested for the durability of their insecticidal activity. A subsample of randomly selected nets from cohort 2 sampled at 12, 24 and 36 months post-distribution were tested in experimental huts at each time point. PermaNet^®^ 3.0 nets obtained from the nearby village of Avokanzoun were marked at baseline in 200 households and collected (and replaced with new nets) at 12, 24 and 36 months post-distribution and tested in the hut trials together with ITNs from the cluster-RCT.

### Experimental hut trial treatments

Experimental hut trials were performed to compare the entomological efficacy of Interceptor^®^, PermaNet^®^ 3.0, Royal Guard^®^ and Interceptor^®^ G2 ITNs withdrawn from households at 12, 24 and 36 months post-distribution. At each annual time point, the efficacy of the field-aged ITNs was compared to new, unused nets of each type and an untreated net as a negative control. A total of 54 replicate field-aged ITNs and 6 new ITNs of each type were tested at each annual time point in 1 or 2 replicate hut trials and rotated within the treatment daily. Before each hut trial, the mean hole index of the field-aged nets was measured for each ITN type following WHO guidelines [17]. To simulate wear-and-tear from routine operational use, all new ITNs and untreated control nets were given 6 holes measuring 4 × 4 cm—two on each of the long side panels and one on each of the short side panels—as per WHO guidelines [17]. The nets were erected inside the experimental huts by tying the edges of the roof panel to nails fixed at the upper corners of the hut wall using string. The following treatment arms were evaluated in each experimental hut trial:

1. Untreated net (control)—6 replicates.
2. Interceptor^®^ (new)—6 replicates.
3. Interceptor^®^ (field-aged 12, 24 or 36 months)—at least 54 replicates.
4. PermaNet^®^ 3.0 (new)—6 replicates.
5. PermaNet^®^ 3.0 (field-aged 12, 24 or 36 months)—at least 54 replicates.
6. Royal Guard^®^ (new)—6 replicates.
7. Royal Guard^®^ (field-aged 12, 24 or 36 months)—at least 54 replicates.
8. Interceptor^®^ G2 (new)—6 replicates.
9. Interceptor^®^ G2 (field-aged 12, 24 or 36 months)—at least 54 replicates.

### Experimental hut trial procedure

The field-aged nets were evaluated in experimental huts the same year they were withdrawn. The hut trials were performed at the same hut site from May to September 2021, April to June 2022 and May to July 2023, with nets withdrawn at 12, 24 and 36 months, respectively. Each trial continued for one full treatment rotation (54 nights over 9 weeks) except for at 12 months, when two consecutive rotations were performed to increase mosquito sample sizes. Treatments were rotated between experimental huts weekly according to a Latin square design to control for the hut position effect, while volunteers were rotated daily to control for differences in individual host attractiveness to mosquitoes. Mosquito collections were performed 6 days per week; on the 7th day, the huts were cleaned and aired to prevent contamination before the next rotation cycle.

### Mosquito collections and processing

Nine (9) consenting human volunteers slept in experimental huts from 21:00 to 06:00 during each trial to attract wild, free-flying mosquitoes. Each morning of the trial, volunteers collected all mosquitoes from the different compartments of the hut (under the net, room, and veranda) and deposited them in labelled plastic cups using a torch and aspirator. Mosquito collections were then transferred to the field laboratory for morphological identification and scoring of immediate mortality (live/dead) and blood-feeding (unfed/blood-fed). Live female mosquitoes identified as species of the *An. gambiae* complex were retained in holding cups and provided access to cotton wool soaked in 10% (w/v) glucose solution. Delayed mortality was recorded every 24 h up to 72 h after collection for all treatments. Mortality after 72 h was used as the primary outcome measure to account for the delayed action of CFP [27] and to provide a single outcome measure for killing effects. To evaluate the impact of Royal Guard^®^ on mosquito reproduction, subsamples of surviving blood-fed mosquitoes were dissected to observe ovary development and score fertility according to Christophers’ stages of egg development [28]. Mosquitoes were classified as fertile if eggs had fully developed to Christophers’ stage V and infertile if eggs had not fully developed and remained at stages I–IV.

### Experimental hut trial outcome measures

The primary outcome measures used to express the efficacy of the experimental hut treatments against pyrethroid-resistant *An. gambiae* and compare the impact of the next-generation ITNs to the pyrethroid-only net—Interceptor^®^—were:

i. Mortality (%)—the proportion of dead mosquitoes 72 h after collection.
ii. Fertility (%)—the proportion of dissected mosquitoes scored fertile.

The secondary outcome measures used to express the efficacy of the experimental hut treatments against pyrethroid-resistant *An. gambiae* were:

i. Entry (*n*)—the total number of mosquitoes collected.
ii. Deterrence (%)—the reduction in entry with the treatment relative to the untreated control. Calculated as follows:
  $$Deterrence \left(\%\right)= \frac{100 (Tu-Tt)}{Tu},$$
  where *Tu* is the number of mosquitoes entering the untreated control and *Tt* is the number of mosquitoes entering the treatment.
iii. Exophily (%)—exiting rates due to the potential irritant effect of treatments expressed as the proportion of mosquitoes collected in the veranda.
iv. . Blood-feeding (%)—the proportion of blood-fed mosquitoes.
v. . Blood-feeding inhibition (%)—the reduction in the proportion of blood-fed mosquitoes in the treatment relative to the untreated control. Calculated as follows: where *Bfu* is the proportion of blood-fed mosquitoes in the untreated control and *Bft* is the proportion of blood-fed mosquitoes in the treatment.
  $$Blood feeding inhibition \left(\%\right)=\frac{100 (Bfu-Bft) }{Bfu}$$
vi. Reduction in fertility (%)—the reduction in the proportion of dissected mosquitoes scored as fertile with the treatment compared to the untreated control. Calculated as follows: where *Fu* is the proportion of fertile mosquitoes in the untreated control and *Ft* is the proportion of fertile mosquitoes in the treatment.
  $$\text{Reduction in fertility }\left(\text{\%}\right)= \frac{100(Fu-Ft)}{Fu}$$

### Monitoring susceptibility of the Covè vector population

To monitor the resistance profile of the Covè vector population over time, WHO tube tests and bottle bioassays were performed in the same year of each experimental hut trial (2021, 2022, 2023) to assess the susceptibility to the AIs in the study ITNs and support interpretation of the results. Mosquitoes were exposed to filter papers treated with the discriminating concentrations of alpha-cypermethrin (0.05%) and deltamethrin (0.05%) in tube tests and to bottles coated with the discriminating concentrations of CFP (100 µg/bottle) and PPF (100 µg/bottle) to assess susceptibility to these insecticides. Pyrethroid resistance intensity was investigated by exposing mosquitoes to 5× (0.25%) and 10x (0.50%) the discriminating concentrations of alpha-cypermethrin and deltamethrin. Finally, PBO synergism and the contribution of overexpressed cytochrome P450 monooxygenases (P450s) to pyrethroid resistance were assessed by pre-exposing mosquitoes to the discriminating concentrations of alpha-cypermethrin (0.05%) and deltamethrin (0.05%) with pre-exposure to PBO (4%). Filter papers used for WHO tube tests were procured from the Universiti Sains Malaysia. Test bottles for WHO bottle bioassays with CFP and PPF were prepared according to WHO guidelines [29].

Mosquitoes used for the bioassays were collected as larvae from breeding sites near the experimental huts and subsequently reared to adulthood. At each time point, at least ~ 100 mosquitoes were exposed to each treatment for 60 min in four replicates of ~ 25 per tube/bottle. Unfed mosquitoes aged 3–5 days were used for pyrethroid and CFP exposures, while for PPF, blood-fed mosquitoes aged 5–7 days were used to facilitate oogenesis and allow for assessment of the impact of PPF on mosquito reproduction. Parallel exposures were performed with filter papers impregnated with silicone oil, PBO (4%) alone and acetone-coated bottles as controls. At the end of exposure, mosquitoes were transferred to untreated containers and provided access to cotton wool soaked in 10% (w/v) glucose solution. Mortality was recorded after 24 h for the pyrethroid exposures and every 24 h up to 72 h for the CFP and PPF exposures. To assess PPF susceptibility, surviving mosquitoes exposed to PPF and the corresponding negative control were dissected after delayed mortality recording to observe ovary development using a compound microscope and score fertility according to Christophers’ stages of egg development [28, 30]. Mosquitoes were classified as fertile if eggs had fully developed to Christophers’ stage V and infertile if eggs had not fully developed and remained at stages I–IV.

### Chemical analysis of net pieces

Net pieces measuring 30 × 30 cm were cut from new and field-aged nets at each annual time point at positions outlined in WHO guidelines [22]. After cutting, the net pieces were labelled, wrapped in aluminium foil, and stored in a refrigerator at 4 ± 2 °C to prevent AI migration in the fabric. The net pieces were then sent to Centre Walloon de Recherches Agronomiques, Belgium, for chemical analysis to measure changes in total AI content over their lifespan. The analytical methods used—which were based on those recommended by the Collaborative International Pesticides Analytical Council—have been described previously [25, 31].

### Data management and analysis

For experimental hut trial data, the total numbers of alive/dead, blood-fed/unfed and fertile/infertile mosquitoes in the different compartments of the hut were pooled with each treatment across the various trials to calculate different proportional outcomes (72 h mortality, blood-feeding, exophily, inside the net, fertility) with corresponding 95% confidence intervals (CIs). Differences between treatments for these proportional binary outcomes were analysed using logistic regression, while differences in count outcomes (entry) were analysed using negative binomial regression. Because two treatment rotations were performed at 12 months and some treatments were tested across multiple trials, the analysis for mosquito entry was adjusted to account for the number of days each treatment was tested. New ITNs were also analysed for each outcome to generate a single estimate across all time points. In addition to the primary explanatory variable of treatment, each model included hut, sleeper, trial period, ITN hole index and day as fixed effects to control for variation associated with differences in individual sleeper and hut attractiveness, seasonality, net condition and overdispersion. The regression analyses generated adjusted odds ratios (ORs) with corresponding 95% CIs, which were used to assess the impact of the next-generation ITNs on the primary outcomes of mosquito mortality and fertility compared to the pyrethroid-only net, Interceptor^®^. P-values derived from the models were also used to assign compact letter displays denoting the statistical significance of all pairwise comparisons at the 5% level for both primary and secondary outcomes. All regression analyses were performed in Stata version 18.

Post-hoc simulation-based power analyses were performed separately after each experimental hut trial using the ‘power_calculator_ITN’ function in R version 4.3.2. These analyses estimated that the power to detect a significant difference in mosquito mortality after 72 h between field-aged Interceptor^®^ and field-aged Interceptor^®^ G2 was 99.8%, 95% CI [99.3, 100] at 12 months, 96.7%, 95% CI [95.4, 97.7] at 24 months and 86.4%, 95% CI [84.1, 88.5] at 36 months.

The susceptibility of the Covè vector population was interpreted based on observed mortality and fertility rates in tube and bottle bioassays according to WHO guidelines [29]. The chemical analysis results demonstrating the total AI content in ITN pieces were used to calculate the proportional AI retention in field-aged nets compared to new nets at each annual time point. All data was recorded by hand on standardized forms before double entry into databases in Microsoft Excel.

### Ethical considerations

Approval for the conduct of the experimental hut trials involving human volunteers was obtained from the ethics review boards of the Ministry of Health in Benin (ref: No. 6/30/MS/DC/DRFMT/CNERS/SA), the London School of Hygiene and Tropical Medicine (LSHTM) (ref: No. 16237), and the WHO (ref: ERC.0003153). Informed written consent was obtained from all human volunteers prior to their participation. All volunteers were offered a free course of chemoprophylaxis to mitigate the risk of malaria infection, and a stand-by nurse was available throughout the trials to assess any volunteers presenting with febrile symptoms or an adverse reaction to the test items.

## Results

### Experimental hut results

Full experimental hut results summarizing the total number of live/dead, unfed/blood-fed and fertile/infertile mosquitoes per treatment arm with descriptive statistics are provided as supplementary material (Table S1).

### Entry, exiting and inside net results of wild, pyrethroid-resistant *An. gambiae*

A total of 21,982 mosquitoes were collected across the three trials. Mosquito deterrence was similar between new and field-aged nets at each time point for Interceptor^®^ and Interceptor^®^ G2 (p > 0.05) (Table 2). In contrast, deterrence was consistently lower with field-aged PermaNet^®^ 3.0 compared to new PermaNet^®^ 3.0 and higher with field-aged Royal Guard^®^ compared to new Royal Guard^®^ (p < 0.05). Between field-aged nets, deterrence was consistently lower with nets withdrawn at 24 and 36 months compared to 12 months. Exiting was 39% with the untreated control net, and exiting was significantly higher with all ITNs regardless of net type and age (p < 0.05). Between new nets, exiting rates were significantly lower with Interceptor^®^ (56%) compared to PermaNet^®^ 3.0 (76%, p < 0.001), Royal Guard^®^ (71%, p < 0.001) and Interceptor^®^ G2 (63%, p < 0.001). Between field-aged next-generation ITNs, exiting rates were ≥ 70% and broadly similar at each time point. Exiting increased significantly with field-aged nets compared to new nets for Interceptor^®^ and Interceptor^®^ G2, while with PermaNet^®^ 3.0 and Royal Guard^®^, exiting rates were broadly similar regardless of age. The proportion of mosquitoes collected inside nets was significantly lower with field-aged nets compared to new nets across all ITN types. The proportion inside field-aged nets was also similarly low across all time points (Table 1).

**Table 1:** Entry, exophily and inside net results of wild, pyrethroid-resistant *Anopheles gambiae* entering experimental huts in Covè, Benin

Net type	Net status	Mean hole index	*N* collected	Mean collected per night*	% Deterrence	*N* inside veranda	% Exophily (95% CIs)	*N* inside net	% Inside net (95% CIs)
Untreated net (control)	–	138.0	3060	14.2^ab^	–	1195	39.1^a^ (37.4–40.8)	1251	40.9^a^ (39.2–42.6)
Interceptor^®^	New	138.0	1991	9.2^cd^	35.2	1115	56.0^b^ (53.8–58.2)	465	23.4^b^ (21.5–25.3)
	12 months	110.6	774	7.2^ef^	49.3	532	68.7^cdef^ (65.4–72.0)	24	3.1^c^ (1.9–4.3)
	24 months	457.9	797	14.8^ab^	− 0.7	549	68.9^cde^ (65.7–72.1)	5	0.6^def^ (0.1–11.1)
	36 months	497.8	599	11.1^cg^	− 4.2	395	65.9^cg^ (62.1–69.7)	1	0.2^def^ (0.0–0.6)
PermaNet^®^ 3.0	New	138.0	1708	7.9^de^	44.4	1304	76.3^defh^ (74.3–78.3)	163	9.5^g^ (8.1–10.9)
	12 months	86.1	795	7.4^ef^	47.9	612	77.0^i^ (74.1–79.9)	11	1.4^d^ (0.6–2.2)
	24 months	320.6	1127	20.9^h^	− 3.5	825	73.2^fhi^ (70.6–75.8)	1	0.1^e^ (0.0–0.3)
	36 months	384.2	682	12.6^ag^	− 47.2	484	71.0^cdefh^ (67.6–74.4)	4	0.6^def^ (0.0–1.2)
Royal Guard^®^	New	138.0	3340	15.5^b^	− 9.2	2378	71.2^c^ (69.7–72.7)	363	10.9^g^ (9.8–12.0)
	12 months	246.9	867	8.0^de^	43.7	676	78.0^hi^ (75.2–80.8)	3	0.3^ef^ (0.0–0.7)
	24 months	528.0	1120	20.7^h^	− 13.4	813	72.6^dfhi^ (70.0–75.2)	2	0.2^ef^ (0.0–0.5)
	36 months	418.1	786	14.6^ab^	− 45.8	603	76.7^cde^ (73.7–79.7)	3	0.4^def^ (0.0–0.8)
Interceptor^®^ G2	New	138.0	2123	9.8^cd^	31.0	1329	62.6^g^ (60.5–64.7)	379	17.9^h^ (16.3–19.5)
	12 months	139.4	627	5.8^f^	59.2	471	75.1^hi^ (71.7–78.5)	1	0.2^ef^ 0.0–0.5)
	24 months	819.8	858	15.9^b^	18.3	598	69.7^cde^ (66.6–72.8)	1	0.1^ef^ (0.0–0.3)
	36 months	598.0	728	13.5^abg^	− 12.0	514	70.6^ce^ (67.3–73.9)	5	0.7^df^ (0.1–1.3)

Data with untreated control and new nets are pooled across different trials to provide a single efficacy estimate

*Values in the same column bearing a common letter do not differ significantly at the 5% level (i.e., p > 0.05) according to regression analysis

### Blood-feeding protection against wild pyrethroid-resistant *An. gambiae*

**Fig. 2:**
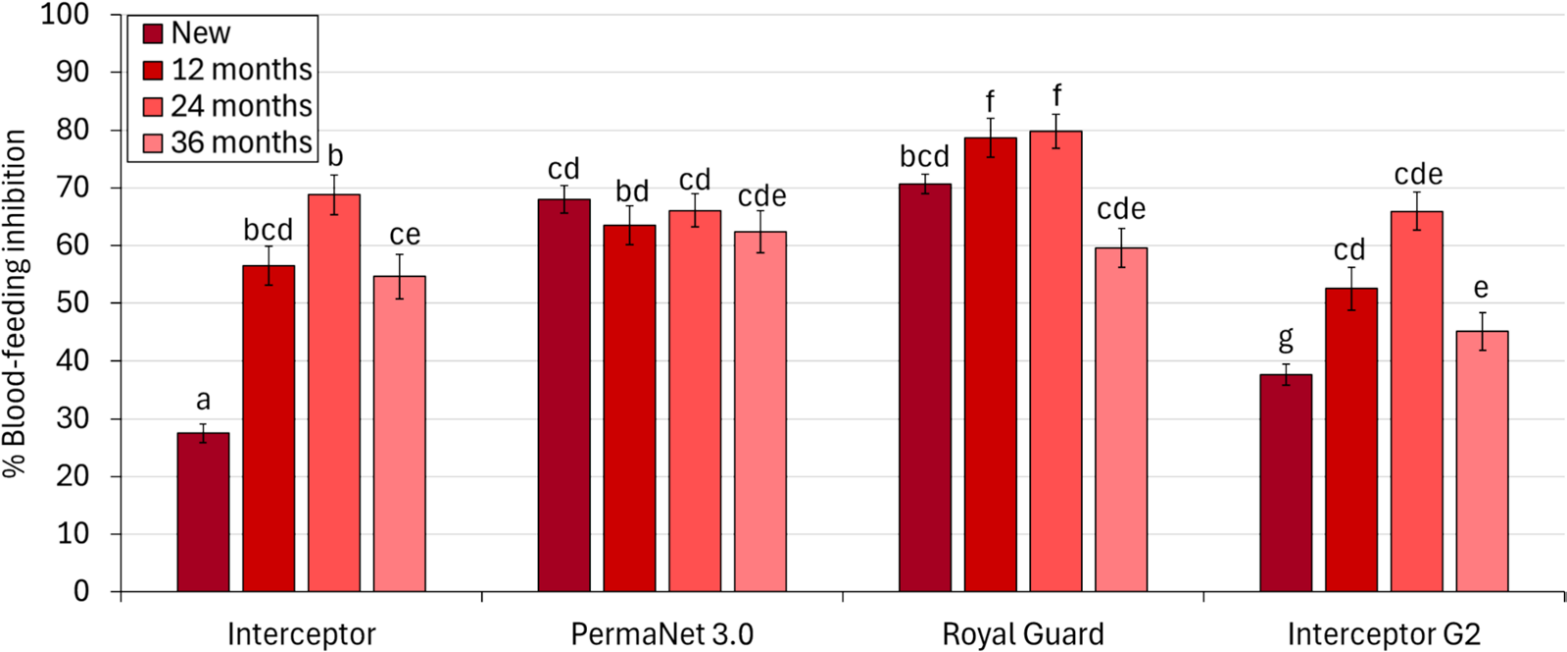
Blood-feeding inhibition of wild pyrethroid-resistant *Anopheles gambiae* entering experimental huts in Covè, Benin. Data with untreated control and new nets are pooled across different trials to provide a single efficacy estimate. Bars bearing a common letter are not significantly different at the 5% level (p > 0.05) according to logistic regression analysis. Error bars represent 95% confidence intervals

The blood-feeding rate with the untreated control net was 63%, and all ITNs inhibited blood-feeding regardless of net type and age (p < 0.001). Between new nets, blood-feeding inhibition was higher for all of the next-generation ITNs compared to Interceptor^®^ (68% with PermaNet^®^ 3.0, 62% with Royal Guard^®^ and 38% with Interceptor^®^ G2 vs 28% with Interceptor^®^, p < 0.001). In comparison, PermaNet^®^ 3.0 and Royal Guard^®^ provided superior blood-feeding protection than Interceptor^®^ G2 (68% and 79% vs 38%, p < 0.001) (Fig. 2). With field-aged nets however, blood-feeding inhibition was generally similar between net types at each time point except at 12 and 24 months when Royal Guard^®^ outperformed the other ITNs (79% with Royal Guard^®^ vs 57% with Interceptor^®^, 64% with PermaNet^®^ 3.0 and 53% with Interceptor^®^ G2 at 12 months, p < 0.001 and 80% with Royal Guard^®^ vs 69% with Interceptor^®^, 66% with PermaNet^®^ 3.0 and 66% with Interceptor^®^ G2 at 24 months, p < 0.05). The mean hole index of field-aged ITNs was generally higher with older nets except with Royal Guard^®^ and Interceptor^®^ G2, where improved fabric integrity was observed with nets withdrawn at 36 months compared to 24 months. Despite the progressive loss in the physical integrity of field-aged nets, blood-feeding inhibition with Interceptor^®^ Royal Guard^®^ and Interceptor^®^ G2 increased steadily from baseline to 24 months before decreasing again at 36 months. In contrast, levels of blood-feeding inhibition with PermaNet^®^ 3.0 were similar across all time points (p > 0.05).

### Mortality rates of wild pyrethroid-resistant *An. gambiae*

**Fig. 3:**
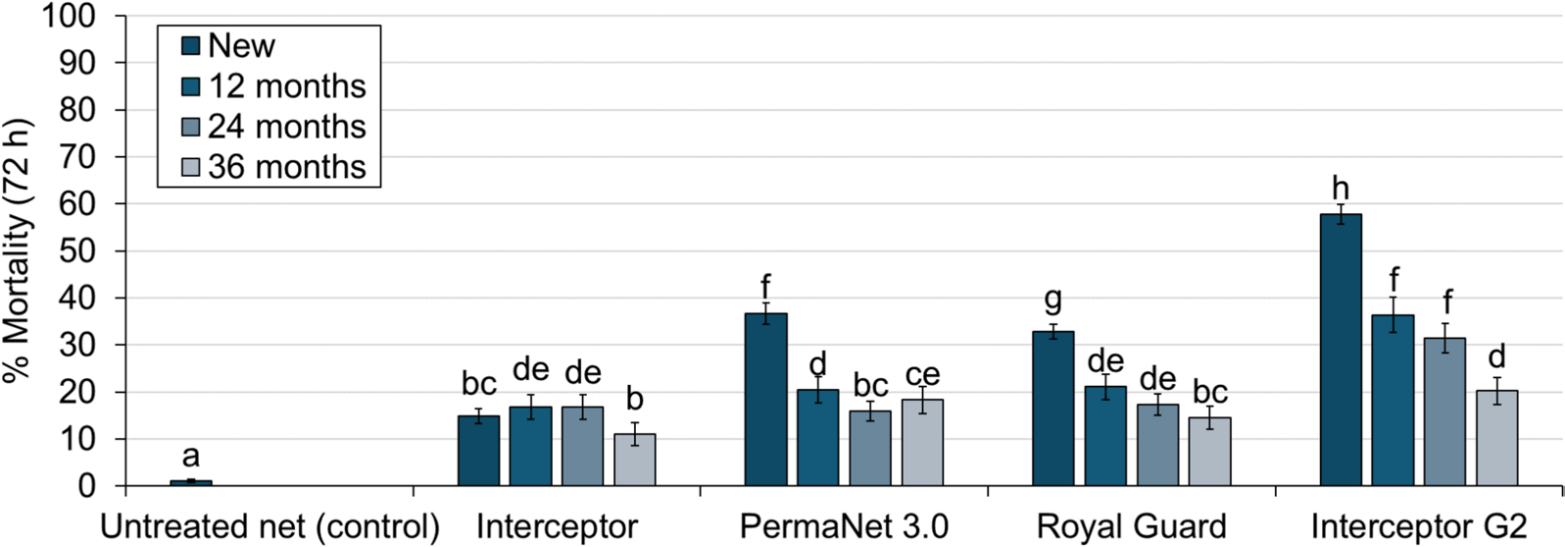
Mortality rates of wild pyrethroid-resistant *Anopheles gambiae* entering experimental huts in Covè, Benin. Data with untreated control and new nets are pooled across different trials to provide a single efficacy estimate. Bars bearing a common letter are not significantly different at the 5% level (p > 0.05) according to logistic regression analysis. Error bars represent 95% confidence intervals

**Fig. 4:**
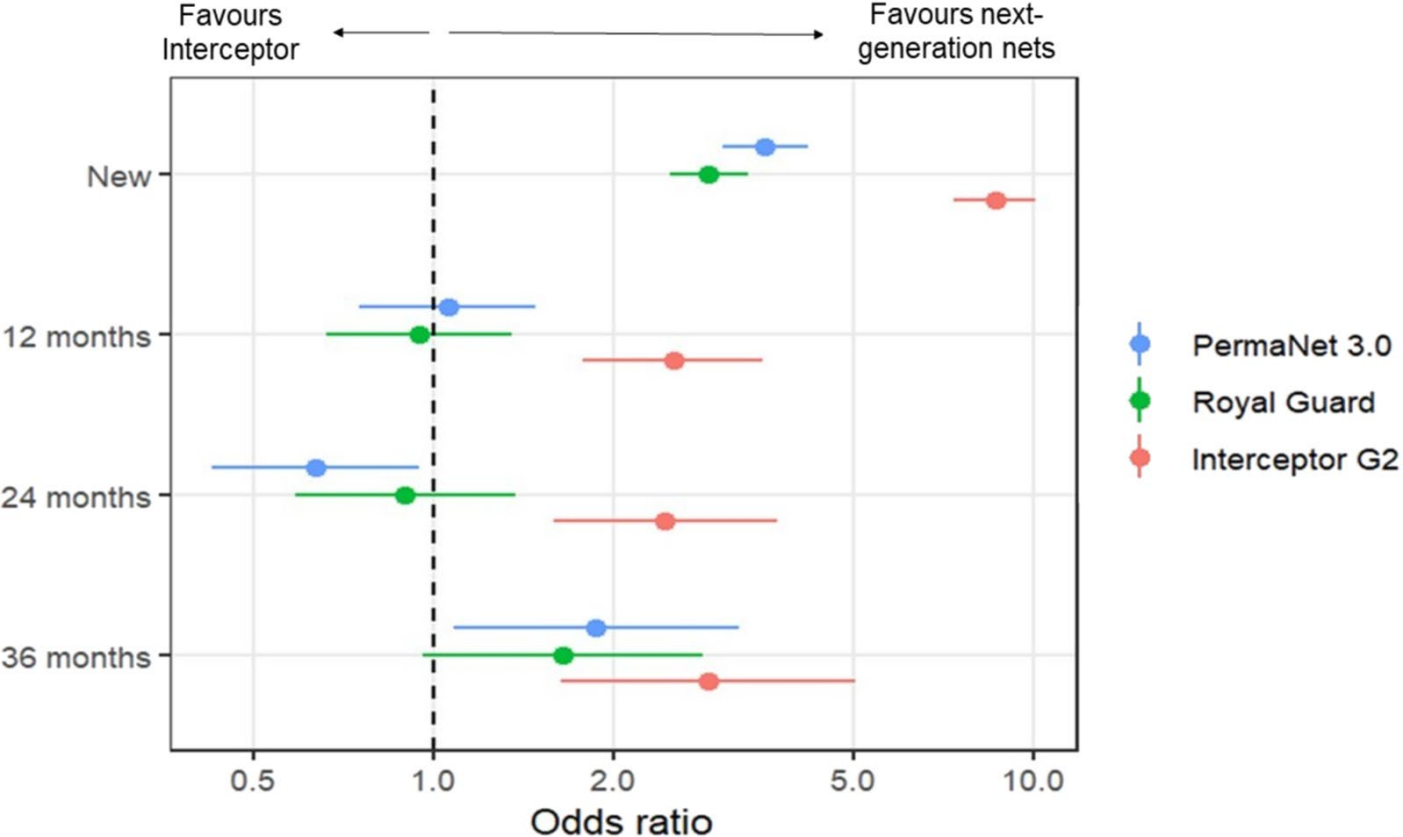
Odds ratios describing the difference in mortality with next-generation nets compared to the pyrethroid-only net. The dashed line represents an odds ratio of 1, indicating no difference in mortality. Odds ratios > 1 indicate higher mortality with next-generation nets. Data with new nets are pooled across different trials to provide a single efficacy estimate. Error bars represent 95% confidence intervals

Mortality with the untreated control net was low (1%), and all ITNs improved mortality regardless of net type and age (p < 0.001). Interceptor^®^ G2 outperformed all study ITNs in terms of mortality, killing significantly greater proportions of mosquitoes than Interceptor^®^, PermaNet^®^ 3.0 and Royal Guard^®^ across all time points (p < 0.05) (Fig. 3). Comparing between new nets across the study showed that the odds of mortality were significantly higher with all next-generation ITNs compared to Interceptor^®^ with PermaNet^®^ 3.0 (OR = 3.57, CIs = 3.03–4.21), Royal Guard^®^ (OR = 2.87, 95% CI [2.48, 3.34]) and Interceptor^®^ G2 (OR = 8.64, 95% CI [7.38, 10.11]) (Fig. 4). With field-aged nets withdrawn at 12 and 24 months, Interceptor^®^ G2 continued to significantly outperform Interceptor^®^ at 12 months (OR = 2.51, 95% CI [1.77, 3.53]) and 24 months (OR = 2.43, 95% CI [1.59, 3.73]) whereas no improvement in mortality was observed with PermaNet^®^ 3.0 at 12 months (OR = 1.05, 95% CI [0.75, 1.48]) and 24 months (OR = 0.63, 95% CI [0.43–0.95]) nor Royal Guard^®^ at 12 months (OR = 0.95, 95% CI [0.66, 1.35]) and at 24 months (OR = 0.90, 95% CI [0.59, 1.37]). At 36 months however, odds of mortality were higher compared to Interceptor^®^ with PermaNet^®^ 3.0 (OR = 1.86, 95% CI [1.08, 3.23]), Royal Guard^®^ (OR = 1.64, 95% CI [0.96, 2.81]) and Interceptor^®^ G2 (OR = 2.87, 95% CI [1.63, 5.05]) with overlapping CIs indicating no substantial difference in mortality odds between net types.

Although Interceptor^®^ produced the lowest mortality rates of all ITNs tested, field-ageing did not negatively affect its impact on vector mortality. Indeed, new Interceptor^®^ induced 12% mortality and with field-aged nets, this improved slightly at 12 months (17%, p = 0.006) and 24 months (17%, p = 0.004) before returning to similar levels as the new nets at 36 months (11%, p = 0.05). In contrast, mortality with the next-generation ITNs decreased progressively with increasing time elapsed from distribution. This reduction was most pronounced with Interceptor^®^ G2, where mortality fell from 58% with new nets to 36% at 12 months (p < 0.001), 31% at 24 months (p < 0.001) and 20% at 36 months (p < 0.001). New PermaNet^®^ 3.0 induced 37% mortality and this also fell significantly to 20% at 12 months (p < 0.001), 16% at 24 months (p < 0.001) and 18% at 36 months (p < 0.001). The trend was remarkably similar with Royal Guard^®^, which induced 33% mortality with new nets before decreasing significantly to 21% at 12 months (p < 0.001), 17% at 24 months (p < 0.001) and 15% at 36 months (p < 0.001).

### Impact on the fertility of wild pyrethroid-resistant *An. gambiae*

**Fig. 5:**
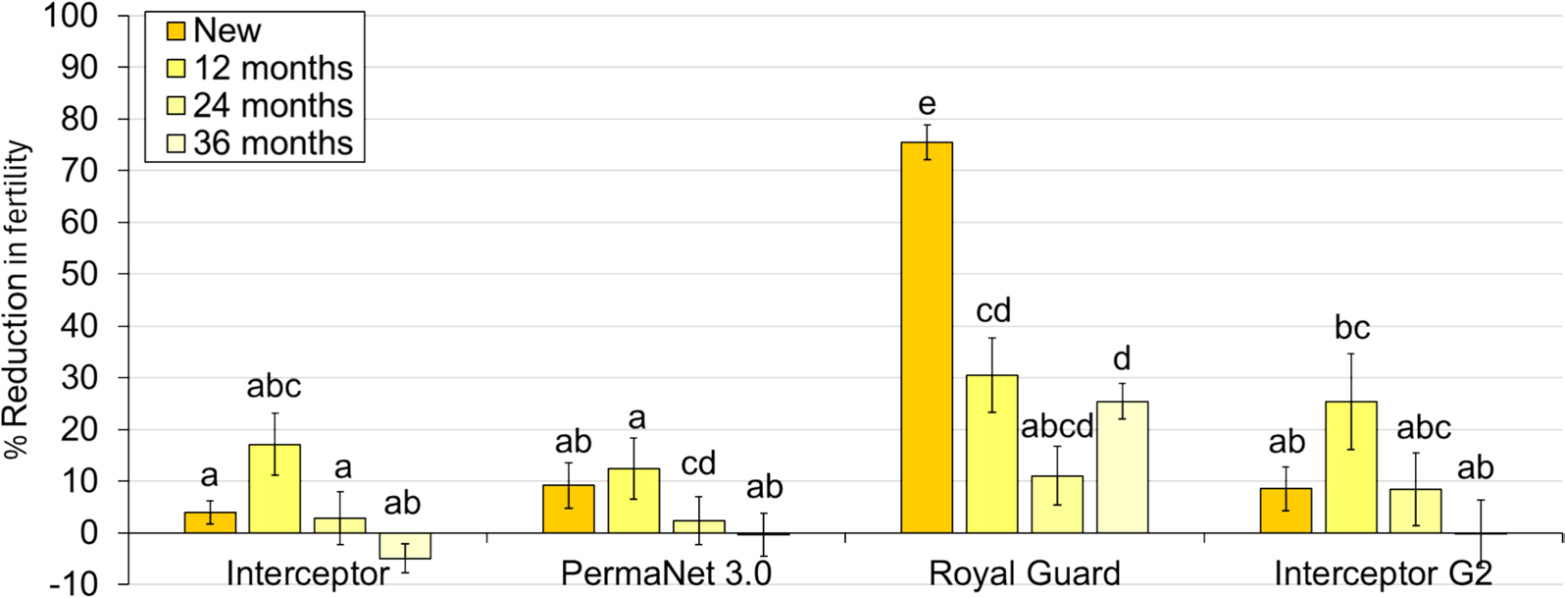
Reduction in fertility of wild pyrethroid-resistant *Anopheles gambiae* entering experimental huts in Covè, Benin. Data with untreated control and new nets are pooled across different trials to provide a single efficacy estimate. Bars bearing a common letter are not significantly different at the 5% level (p > 0.05) according to logistic regression analysis. Error bars represent 95% confidence intervals

**Fig. 6:**
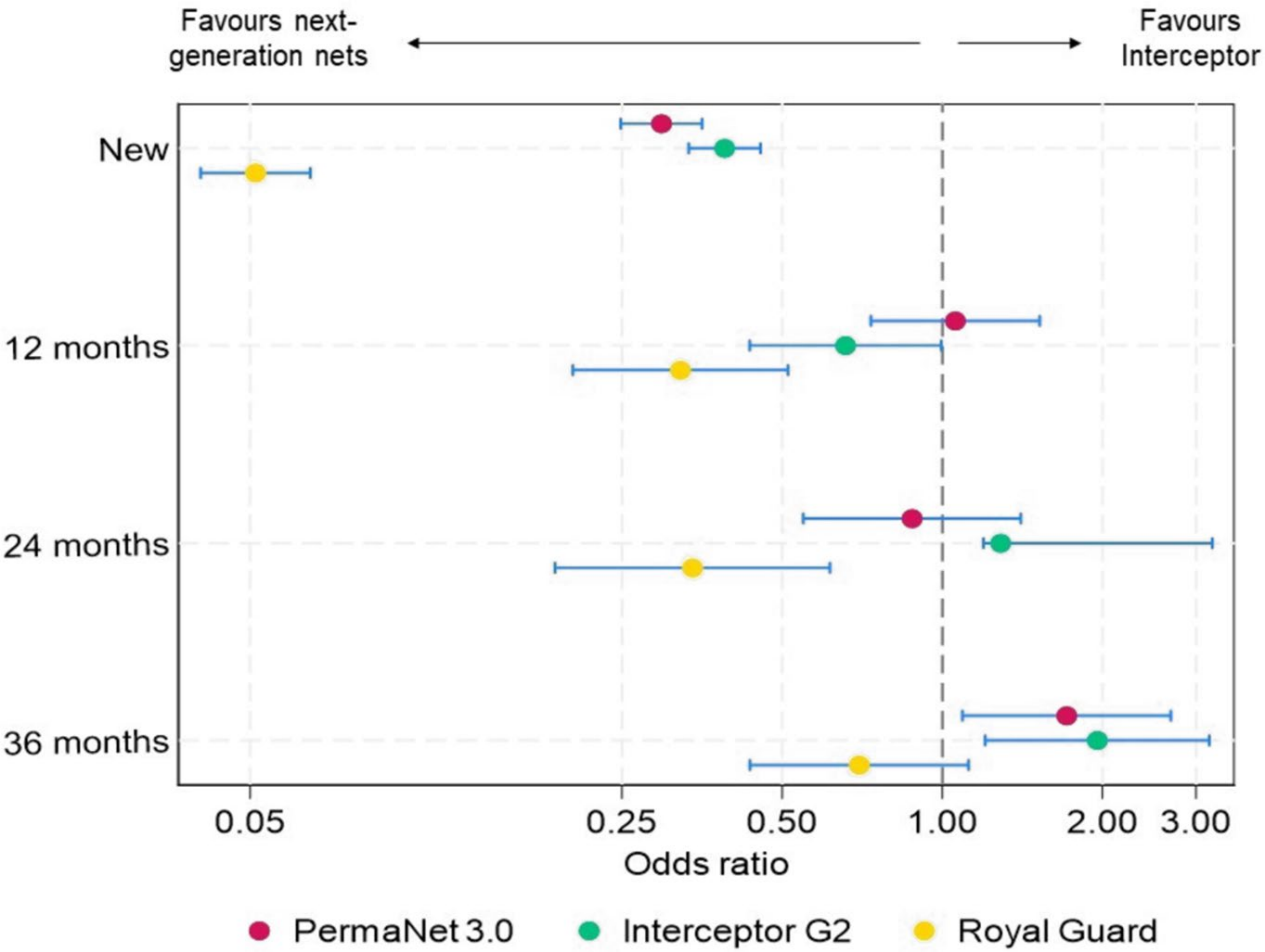
Odds ratios describing difference in fertility rates with next-generation nets compared to the pyrethroid-only net. The dashed line represents an odds ratio of 1, indicating no difference in fertility. Odds ratios < 1 indicate a greater reduction in fertility with next-generation nets. Data with new nets are pooled across different trials to provide a single efficacy estimate. Error bars represent 95% confidence intervals

The overall fertility rate with the untreated control net was 92%. Royal Guard^®^ arms consistently reduced fertility relative to the control and while some of the other ITNs also significantly reduced fertility compared to the control, the effect size small with statistical significance varying across arms. At each time point, Royal Guard^®^ also induced a significantly higher reduction in fertility than the other ITNs except at 24 months when there was no significant difference between study arms (11% with Royal Guard^®^ vs 3% with Interceptor p = 0.08, 11% with Royal Guard^®^ vs 2% with PermaNet^®^ 3.0 p = 0.55 and 11% with Royal Guard^®^ vs 8% with Interceptor^®^ G2 p = 0.80) (Fig. 5). With new nets, the odds of fertility with Royal Guard^®^ were very low compared to Interceptor^®^ (OR = 0.05, 95% CI [0.04, 0.06]); however, this increased significantly at 12 months (OR = 0.32, 95% CI [0.20, 0.51]), 24 months (OR = 0.34, 95% CI [0.19, 0.61]) and 36 months (OR = 0.70, 95% CI [0.43, 1.12]) (Fig. 6), demonstrating a loss in its sterilizing effect. There was no significant difference in fertility across time points for field-aged Royal Guard^®^ (p > 0.05).

### Monitoring susceptibility of the Covè vector population results

WHO tube tests and bottle bioassays were performed in the same year of each experimental hut trial (2021, 2022, 2023) to assess longitudinal changes in the insecticide resistance profile of the Covè vector population and support the interpretation of the results. *An. gambiae* mosquitoes used for the tests were collected as larvae from breeding sites near the experimental huts and reared to adulthood.

**Fig. 7:**
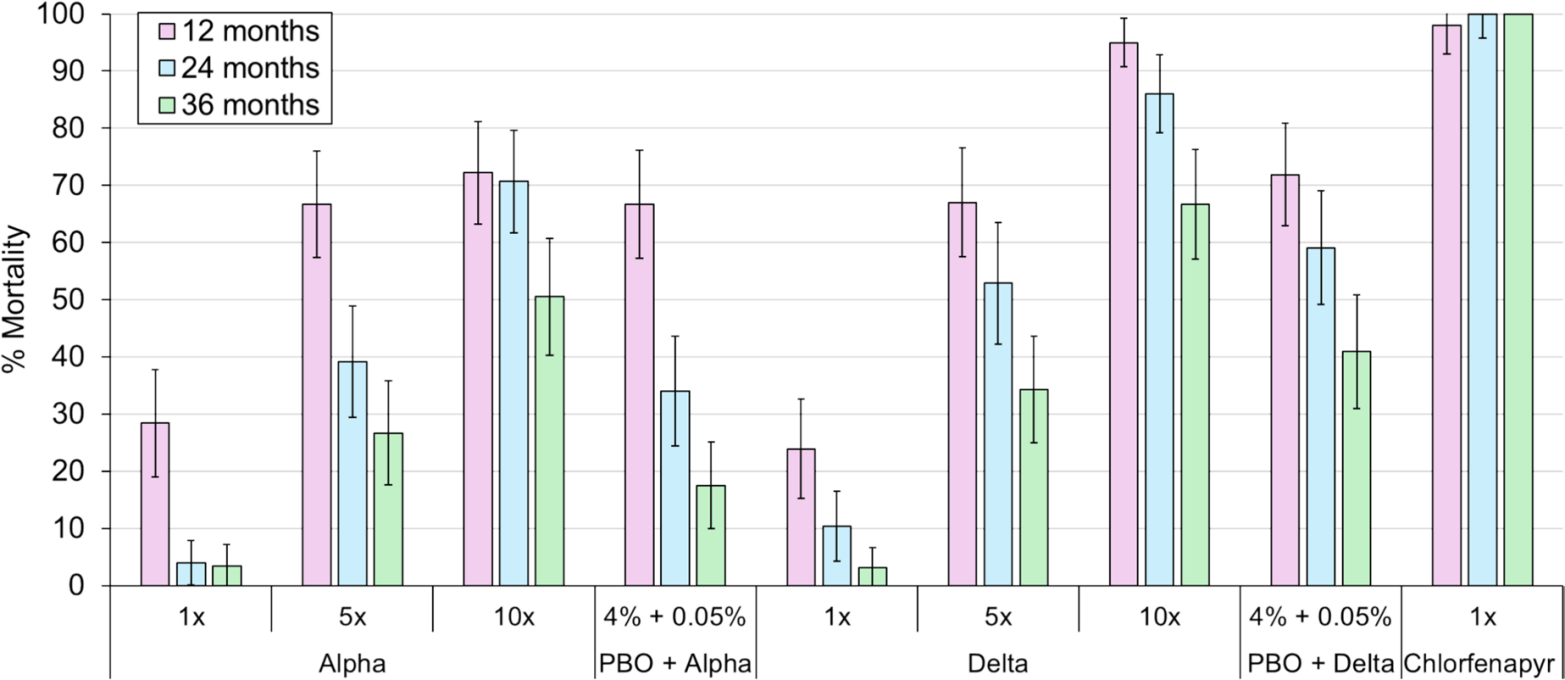
Mortality of field-collected *Anopheles gambiae* in World Health Organization susceptibility bioassays. Tube tests and bottle bioassays were performed during each trial using adult mosquitoes collected as larvae from breeding sites near the experimental huts. Mortality was recorded after 24 h for pyrethroid exposures and after 72 h for chlorfenapyr. Error bars represent 95% confidence intervals

The discriminating concentrations of alpha-cypermethrin and deltamethrin induced low mortality (alpha-cypermethrin: 3–24%, deltamethrin: 3–28%) at all time points, demonstrating the high frequency of pyrethroid resistance in the Covè vector population (Fig. 7). Mortality rates were higher with 5 × and 10 × the discriminating concentrations with both pyrethroids at each time point but still failed to pass susceptibility thresholds (≥ 98%), providing evidence of high-intensity pyrethroid resistance. PBO pre-exposure improved the mortality response to the discriminating concentrations of alpha-cypermethrin and deltamethrin at each time point but failed to restore susceptibility, implicating partial involvement of P450s in pyrethroid resistance. Mortality with pyrethroids, both alone and with PBO pre-exposure, decreased progressively and substantially between 2021 and 2023, suggesting that the frequency and intensity of pyrethroid resistance increased over this period. In contrast, the discriminating concentration of CFP induced high mortality (≥ 98%) at all time points, demonstrating the continued susceptibility of the Covè vector population to pyrroles. Lastly, the reduction in fertility with mosquitoes exposed to the discriminating concentration of PPF was > 92% at all time points, indicating that the vector population was largely susceptible to the insect growth regulator (Table 2). Mortality with the negative controls was low (< 5%), while fertility rates (> 85%) were high, thus validating the tests.

**Table 2:** Fertility rates of field-collected *Anopheles gambiae* in World Health Organization pyriproxyfen susceptibility bioassays

Treatment	Acetone (control)			Pyriproxyfen (100 µg) (1×)		
Time point	2021	2022	2023	2021	2022	2023
*N* exposed	68	200	95	100	328	88
*N* dissected	68	94	87	95	132	70
*N* fertile	60	94	86	7	1	4
% Fertility (95% CIs)	88.2 (80.5–95.9)	100 (–)	98.9 (96.7–100)	7.4 (2.1–12.7)	0.8 (0.0–2.3)	5.7 (0.3–11.1)
% Reduction in fertility	–	–	–	91.6	99.2	94.2

Bottle bioassays with the discriminating concentration of pyriproxyfen (100 µg) were performed during each trial using blood-fed adult mosquitoes collected as larvae from breeding sites near the experimental huts. Mortality was recorded up to 72 h, and a subsample of surviving mosquitoes was dissected to observe ovary development and score fertility.

### Chemical analysis results

**Fig. 8:**
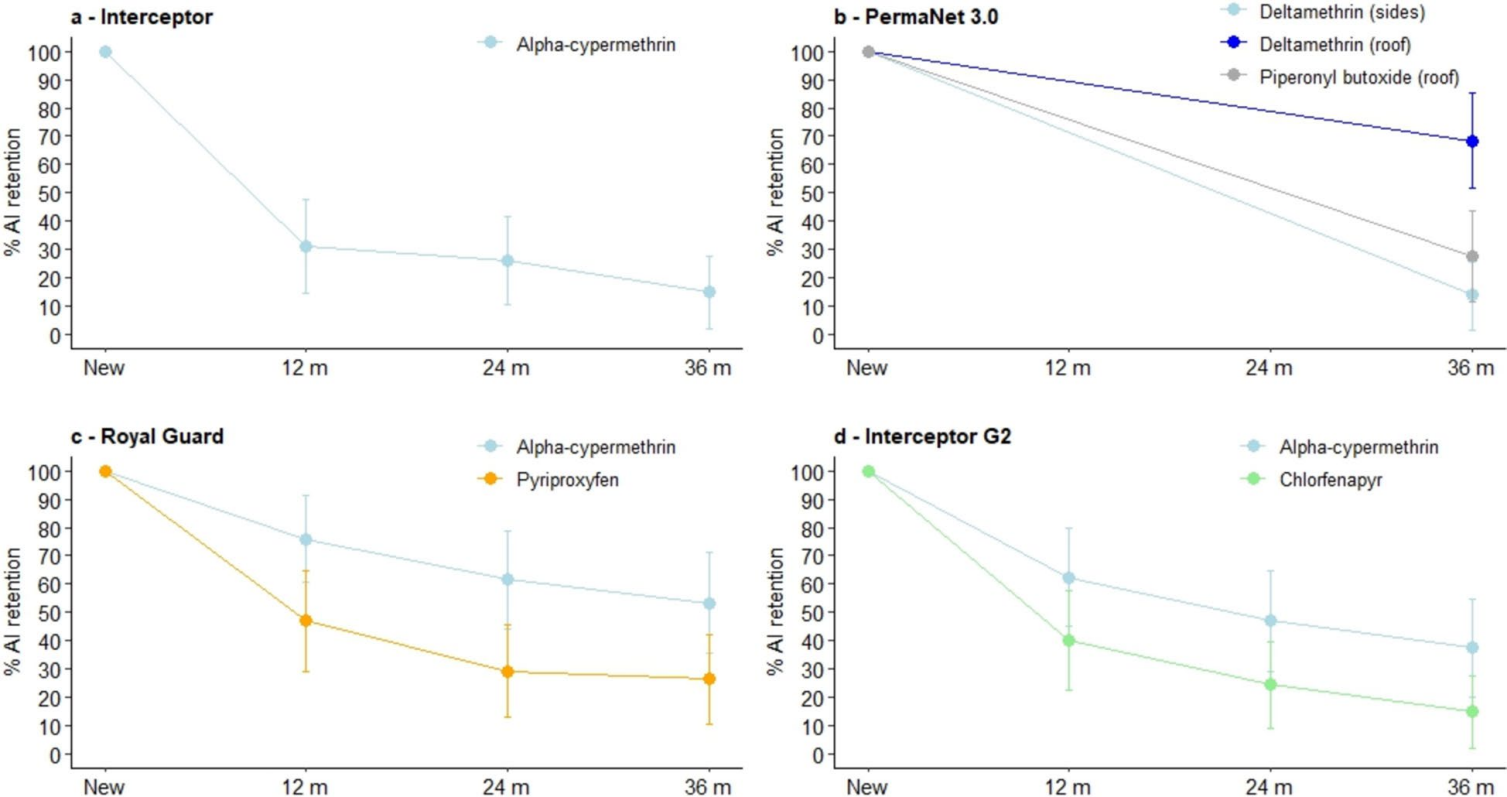
Proportional active ingredient retention in field-aged nets withdrawn at 12, 24 and 36 months post-distribution. Results for Interceptor^®^, PermaNet^®^ 3.0, Royal Guard^®^ and Interceptor^®^ G2 are presented in panels a, b and c and d, respectively. Due to funding constraints, chemical analysis was not performed with PermaNet^®^ 3.0 at 12 and 24 months. Error bars represent 95% confidence intervals

The chemical content of new ITNs was within acceptable margins (± 25%) of specifications declared by the manufacturers. With field-aged nets, proportional AI retention compared to new nets decreased progressively with increasing time elapsed from distribution for all ITN types. The proportional retention of pyrethroid content with field-aged nets withdrawn after 36 months relative to new nets was lowest with alpha-cypermethrin on Interceptor^®^ (15%) and deltamethrin on the side panels of PermaNet^®^ 3.0 (14%) (Fig. 8). In contrast, pyrethroid retention was comparatively higher with deltamethrin on the roof of PermaNet^®^ 3.0 (68%) and with alpha-cypermethrin in Royal Guard^®^ (53%) and Interceptor^®^ G2 (37%). The overall reduction in the content of the partner compound in all next-generation ITNs was high. Proportional AI retention with field-aged nets withdrawn at 36 months compared to new nets was just 27% for PBO in the roof of PermaNet^®^ 3.0, 26% for PPF in Royal Guard^®^ and 15% with CFP in Interceptor^®^ G2. Full summary chemical analysis results are provided as supplementary material (Table S2).

## Discussion

This study assessed the experimental hut efficacy of new and field-aged next-generation ITNs withdrawn at 12, 24 and 36 months from communities in Benin to detect changes in their insecticidal activity against wild malaria vectors over time as part of a larger ITN durability evaluation [21]. WHO ITN durability guidelines do not currently cover experimental hut trials of field-aged ITNs. Nevertheless, by simulating the natural behavioural interactions between host-seeking mosquitoes and nets inside households, these assays may provide more informative data regarding the operational efficacy of ITNs over their intended lifespan than traditional bioassay techniques. The vector population at the Covè hut site was suitable for evaluating the three different next-generation ITN types, showing a high intensity of pyrethroid resistance with some restoration of susceptibility after PBO pre-exposure and susceptibility to CFP and PPF. This resistance profile is broadly consistent with findings from the resistance monitoring component of the cluster-RCT [32].

Mortality rates of wild, pyrethroid-resistant *An. gambiae* entering experimental huts were consistently higher with Interceptor^®^ G2 than with the other ITNs, demonstrating the superiority of pyrethroid-CFP nets over other net classes in this area. Recent experimental hut trials in Benin [31], Cameroon [33], Côte d’Ivoire [34] and Tanzania [35] have also shown that pyrethroid-CFP nets induced higher levels of mortality than pyrethroid-only, pyrethroid-PBO and pyrethroid-PPF nets. The findings corroborate results from cluster-RCTs in Benin [10, 16] and Tanzania [11, 36], as well as observational studies conducted as part of pilot distributions of next-generation nets [37] that showed a greater improvement in malaria control relative to pyrethroid-only nets with pyrethroid-CFP nets compared to pyrethroid-PBO and pyrethroid-PPF nets. It also supports WHO’s strong recommendation for large-scale deployment of pyrethroid-CFP nets over pyrethroid-only nets [13] and their prioritization over other ITN types [38]. Despite the superior performance of Interceptor^®^ G2, mortality rates with this ITN decreased progressively at each annual time point, and at 36 months, the OR describing the difference in mortality compared to Interceptor^®^ did not differ significantly from PermaNet^®^ 3.0 and Royal Guard^®^. A recent secondary analysis [16] of the cluster-RCT in Benin found that the superior epidemiological impact of Interceptor^®^ G2 relative to Interceptor^®^ observed during the first 2 years of the trial [10] was not detected statistically in the 3rd year. The present study provides context for these results and suggests that the dwindling CFP content after 36 months (< 20% retention) and the associated reduction in entomological efficacy may have contributed to the lack of epidemiological benefit observed with Interceptor^®^ G2 in the 3rd year of the Benin trial. Efforts should thus be made to improve the durability of CFP in pyrethroid-CFP nets. Additionally, control programmes could sustain effective coverage in the 3rd year through continuous distribution channels or consider more frequent distribution cycles where resources allow.

Improved mortality rates with PermaNet^®^ 3.0 compared to Interceptor^®^ were only evident with new nets, suggesting that its benefit over pyrethroid-only nets is short-lived compared to Interceptor^®^ G2. Although PermaNet^®^ 3.0 has a higher loading dose of deltamethrin compared to its pyrethroid-only counterpart, PermaNet^®^ 2.0 [26], the reduced efficacy of PermaNet^®^ 3.0 after field-ageing is probably due more to a loss in PBO content over time. The chemical content of PermaNet^®^ 3.0 was not measured at 12 and 24 months due to funding constraints; however, a more significant loss in AI was observed at 36 months for PBO compared to deltamethrin. Given that the inhibitory action of PBO against detoxifying P450 enzymes is known to improve the toxicity of pyrethroids [6], a reduction in its content over time will likely lead to reduced efficacy. Previous studies have also shown significant reductions in the insecticidal activity of pyrethroid-PBO nets within 2 years of distribution associated with poor PBO durability [15]. Developing pyrethroid-PBO net brands with extended insecticidal durability will help improve the viability of this net class for malaria control.

While new PermaNet^®^ 3.0 killed higher proportions of mosquitoes than new Interceptor^®^, the absolute difference in mortality rates was minor. Previous studies in Benin [9, 39] and other areas of West and Central Africa [40, 41] have also demonstrated a modest killing effect of pyrethroid-PBO nets in experimental huts [9, 39–41] and a failure of PBO to fully restore pyrethroid susceptibility in synergist bioassays [42, 43]. This contrasts with findings from East Africa, where the impact of these nets on vector mortality is generally greater [44, 45]. This difference is potentially due to the higher intensity of pyrethroid resistance encountered in the West and Central regions and the presence of more complex resistance mechanisms unaffected by the synergistic action of PBO. Indeed, the susceptibility bioassays performed during these trials provided evidence for an escalation in pyrethroid resistance intensity in the Covè vector population over time and an increasing inability of PBO to restore susceptibility. Experimental hut trials in Cameroon have also documented reductions in the efficacy of pyrethroid-PBO nets associated with a mutation in the glutathione S-transferase epsilon 2 gene (*GSTe2*) [41]. The epidemiological evidence used to inform WHO’s conditional recommendation for pyrethroid-PBO nets [13] was generated exclusively in East African countries [7, 8, 11], and there remains a lack of high-quality data demonstrating the public health value of these nets in other regions. An ongoing cluster-RCT evaluating the impact of pyrethroid-PBO nets on malaria incidence in Côte d’Ivoire will contribute important evidence in this area. But, epidemiological follow-up will only continue for 1 year [46]. The short-lived impact of PermaNet^®^ 3.0 in the present study underlines the need to generate long-term data on the epidemiological efficacy of pyrethroid-PBO nets in West and Central Africa to better inform decision-making in these areas.

The impact of Royal Guard^®^ on mosquito reproduction and mortality was most evident with new nets and declined substantially after field-ageing. The improved killing effect of Royal Guard^®^ over Interceptor^®^ between new nets was probably due to the higher loading dose and surface availability of alpha-cypermethrin and potential longevity-shortening effects of PPF [47, 48]. The impact of new Royal Guard^®^ on mosquito fertility, meanwhile, was attributable to its PPF component, which is known to disrupt ovary development in mosquitoes by mimicking the action of juvenile hormone [49]. The subsequent reduction in the sterilizing effects with field-aged Royal Guard^®^ was thus linked to the rapid loss in PPF content as demonstrated by the chemical analysis results. A previous experimental hut trial conducted at the same site in Benin provided similar evidence for the poor durability of Royal Guard^®^ by showing significant reductions in its sterilizing effects and PPF content after washing 20 times [25]—a process used to simulate the loss of AI over 3 years of operational use. Results from recent cluster-RCTs have also shown that Royal Guard^®^ had no significant impact on malaria incidence or prevalence compared to Interceptor^®^ over several years in both Benin [10, 16] and Tanzania [11, 36]. The present study suggests that these results may partly be due to the rapid loss of PPF and its biological activity in Royal Guard^®^, emphasizing the need to improve the durability of partner compounds in next-generation ITNs. In addition to durability issues, other factors may explain why Royal Guard^®^ failed to improve disease control in the cluster-RCT in Benin. Laboratory studies have revealed that mosquito P450s associated with pyrethroid resistance, including those which are overexpressed in the Covè vector population like CYP6P3 [23], can metabolize PPF in vitro [50] and in heterologous expression systems [51]. Thus, elevated P450 activity may have compromised the performance of Royal Guard^®^. However, given that the reduction in fertility observed in PPF susceptibility bioassays was still relatively high (≥ 90%), further studies are needed to confirm this.

Levels of blood-feeding inhibition increased progressively at each annual time point before falling substantially at 36 months with all ITN types except PermaNet^®^ 3.0. The personal protective benefit of ITNs is attributable to their physical barrier and the excitorepellent properties of the pyrethroid. The improved blood-feeding inhibition with field-aged nets is thus unusual, given that the nets' fabric integrity and total pyrethroid content decreased with increasing time elapsed from distribution. Exiting rates were also generally higher, albeit only slightly, with field-aged nets compared to new nets, suggesting increased excitorepellency following operational use. This finding could be due to contamination of ITNs with repellent products or substances inside households or changes in the dynamics of the pyrethroids in the netting fibres during household use, increasing their surface availability. However, the same trend was not observed with PermaNet^®^ 3.0, which induced similar levels of blood-feeding inhibition across all time points. Further studies are thus needed to explain these findings.

## Conclusions

This study evaluated the bioefficacy of operationally-aged ITNs in experimental huts to detect changes in their insecticidal activity against wild free-flying malaria vectors over time. The findings complement recent cluster-RCT results demonstrating the impact of these ITNs against clinical malaria. Interceptor^®^ G2 outperformed Interceptor^®^, PermaNet^®^ 3.0 and Royal Guard^®^ in terms of mosquito mortality at all time points, confirming the superiority of pyrethroid-CFP nets over other ITN types. Despite this, the improvement was less evident at 36 months due to reduced CFP content. This helps explain recent cluster-RCT results in Benin showing a loss in the epidemiological benefit of Interceptor^®^ G2 over Interceptor^®^ in the 3rd year post-distribution. Between new nets, PermaNet^®^ 3.0 and Royal Guard^®^ demonstrated superior entomological efficacy than Interceptor^®^. However, this improvement was largely lost after field-ageing due to the rapid loss of PBO and PPF in these ITNs and potential tolerance to these compounds in the Covè vector population. All next-generation ITNs failed to maintain an effective level of entomological efficacy over 3 years, and thus, efforts are needed to enhance the durability of the non-pyrethroid compounds on ITNs. Further studies are also required to understand the increase in blood-feeding inhibition observed with some study ITNs after field-ageing and investigate mechanisms of potential resistance to PBO and PPF.

## Supplementary Information

Supplementary Material 1

Supplementary Material 2

## Data Availability

All data supporting the findings of this study are available within the paper and its supplementary material.

## Abbreviations

AI: Active ingredient
CFP: Chlorfenapyr
ITN: Insecticide-treated net
*Kdr*: Knockdown resistance
LSHTM: London School of Hygiene & Tropical Medicine
PBO: Piperonyl butoxide
PPF: Pyriproxyfen
P450: Cytochrome P450 monooxygenase
RCT: Randomized controlled trial
WHO: World Health Organization

## References

[1] Alliance for Malaria Prevention Net Mapping Project. Global Delivery Report 2024. Available from: https://allianceformalariaprevention.com/net-mapping-project/.

[2] Bhatt S, Weiss D, Cameron E, Bisanzio D, Mappin B, Dalrymple U, et al. The effect of malaria control on *Plasmodium falciparum* in Africa between 2000 and 2015. Nature. 2015;526:207–11. 26375008 10.1038/nature15535 PMC4820050

[3] Hancock PA, Hendriks CJ, Tangena J-A, Gibson H, Hemingway J, Coleman M, et al. Mapping trends in insecticide resistance phenotypes in African malaria vectors. PLoS Biol. 2020;18: e3000633. 32584814 10.1371/journal.pbio.3000633 PMC7316233

[4] WHO. World Malaria Report 2023. Geneva: World Health Organization; 2023.

[5] WHO. Global technical strategy for malaria 2016–2030. Geneva: World Health Organization; 2015.

[6] Bingham G, Strode C, Tran L, Khoa PT, Jamet HP. Can piperonyl butoxide enhance the efficacy of pyrethroids against pyrethroid-resistant *Aedes aegypti*? Trop Med Int Health. 2011;16:492–500. 21324051 10.1111/j.1365-3156.2010.02717.x

[7] Protopopoff N, Mosha JF, Lukole E, Charlwood JD, Wright A, Mwalimu CD, et al. Effectiveness of a long-lasting piperonyl butoxide-treated insecticidal net and indoor residual spray interventions, separately and together, against malaria transmitted by pyrethroid-resistant mosquitoes: a cluster, randomised controlled, two-by-two factorial design trial. Lancet. 2018;391:1577–88. 29655496 10.1016/S0140-6736(18)30427-6 PMC5910376

[8] Staedke SG, Gonahasa S, Dorsey G, Kamya MR, Maiteki-Sebuguzi C, Lynd A, et al. Effect of long-lasting insecticidal nets with and without piperonyl butoxide on malaria indicators in Uganda (LLINEUP): a pragmatic, cluster-randomised trial embedded in a national LLIN distribution campaign. Lancet. 2020;395:1292–303. 32305094 10.1016/S0140-6736(20)30214-2 PMC7181182

[9] Ngufor C, Fagbohoun J, Agbevo A, Ismail H, Challenger JD, Churcher TS, et al. Comparative efficacy of two pyrethroid-piperonyl butoxide nets (Olyset Plus and PermaNet 3.0) against pyrethroid resistant malaria vectors: a non-inferiority assessment. Malar J. 2022;21:20. 35016676 10.1186/s12936-022-04041-9 PMC8753866

[10] Accrombessi M, Cook J, Dangbenon E, Yovogan B, Akpovi H, Sovi A, et al. Efficacy of pyriproxyfen-pyrethroid long-lasting insecticidal nets (LLINs) and chlorfenapyr-pyrethroid LLINs compared with pyrethroid-only LLINs for malaria control in Benin: a cluster-randomised, superiority trial. Lancet. 2023;401:435–46. 36706778 10.1016/S0140-6736(22)02319-4

[11] Mosha JF, Kulkarni MA, Lukole E, Matowo NS, Pitt C, Messenger LA, et al. Effectiveness and cost-effectiveness against malaria of three types of dual-active-ingredient long-lasting insecticidal nets (LLINs) compared with pyrethroid-only LLINs in Tanzania: a four-arm, cluster-randomised trial. Lancet. 2022;399:1227–41. 35339225 10.1016/S0140-6736(21)02499-5 PMC8971961

[12] Tiono AB, Ouédraogo A, Ouattara D, Bougouma EC, Coulibaly S, Diarra A, et al. Efficacy of Olyset Duo, a bednet containing pyriproxyfen and permethrin, versus a permethrin-only net against clinical malaria in an area with highly pyrethroid-resistant vectors in rural Burkina Faso: a cluster-randomised controlled trial. Lancet. 2018;392:569–80. 30104047 10.1016/S0140-6736(18)31711-2

[13] WHO. Guidelines for malaria. Geneva: World Health Organization; 2023.

[14] Gichuki PM, Kamau L, Njagi K, Karoki S, Muigai N, Matoke-Muhia D, et al. Bioefficacy and durability of Olyset^®^ Plus, a permethrin and piperonyl butoxide-treated insecticidal net in a 3-year long trial in Kenya. Infect Dis Poverty. 2021;10:135. 34930459 10.1186/s40249-021-00916-2 PMC8691082

[15] Mechan F, Katureebe A, Tuhaise V, Mugote M, Oruni A, Onyige I, et al. LLIN Evaluation in Uganda Project (LLINEUP): the fabric integrity, chemical content and bioefficacy of long-lasting insecticidal nets treated with and without piperonyl butoxide across two years of operational use in Uganda. Curr Res Parasitol Vector Borne Dis. 2022;2: 100092. 35734077 10.1016/j.crpvbd.2022.100092 PMC9207544

[16] Accrombessi M, Cook J, Dangbenon E, Sovi A, Yovogan B, Assongba L, et al. Effectiveness of pyriproxyfen-pyrethroid and chlorfenapyr-pyrethroid long-lasting insecticidal nets (LLINs) compared with pyrethroid-only LLINs for malaria control in the third year post-distribution: a secondary analysis of a cluster-randomised controlled trial in Benin. Lancet Infect Dis. 2024;24:619–28. 38401551 10.1016/S1473-3099(24)00002-1

[17] WHO. Guidelines for laboratory and field-testing of long-lasting insecticidal nets. Geneva: World Health Organization; 2013. Report No. 9241505273.

[18] Lees RS, Armistead JS, Azizi S, Constant E, Fornadel C, Gimnig JE, et al. Strain characterisation for measuring bioefficacy of ITNs treated with two active ingredients (Dual-AI ITNs): developing a robust protocol by building consensus. Insects. 2022;13:434. 35621770 10.3390/insects13050434 PMC9144861

[19] Churcher TS, Stopard IJ, Hamlet A, Dee DP, Sanou A, Rowland M, et al. Projecting epidemiological benefit of pyrethroid-pyrrole insecticide treated nets against malaria. Lancet (preprint). 2023. Available at SSRN: https://papers.ssrn.com/sol3/papers.cfm?abstract_id=4569154. 10.1016/S2214-109X(24)00329-2 PMC11584316 39577971

[20] Sherrard-Smith E, Ngufor C, Sanou A, Guelbeogo MW, N’Guessan R, Elobolobo E, et al. Inferring the epidemiological benefit of indoor vector control interventions against malaria from mosquito data. Nat Commun. 2022;13:3862. 35790746 10.1038/s41467-022-30700-1 PMC9256631

[21] Ngufor C, Fongnikin A, Fagbohoun J, Agbevo A, Syme T, Ahoga J, et al. Evaluating the attrition, fabric integrity and insecticidal durability of two dual active ingredient nets (Interceptor^®^ G2 and Royal^®^ Guard): methodology for a prospective study embedded in a cluster randomized controlled trial in Benin. Malar J. 2023;22:276. 37716970 10.1186/s12936-023-04708-x PMC10504698

[22] Accrombessi M, Cook J, Ngufor C, Sovi A, Dangbenon E, Yovogan B, et al. Assessing the efficacy of two dual-active ingredients long-lasting insecticidal nets for the control of malaria transmitted by pyrethroid-resistant vectors in Benin: study protocol for a three-arm, single-blinded, parallel, cluster-randomized controlled trial. BMC Infect Dis. 2021;21:194. 33607958 10.1186/s12879-021-05879-1 PMC7892705

[23] Ngufor C, N’Guessan R, Fagbohoun J, Subramaniam K, Odjo A, Fongnikin A, et al. Insecticide resistance profile of *Anopheles gambiae* from a phase II field station in Cove, southern Benin: implications for the evaluation of novel vector control products. Malar J. 2015;14:464. 26581678 10.1186/s12936-015-0981-z PMC4652434

[24] Syme T, Nounagnon J, N’Dombidjé B, Gbegbo M, Agbevo A, Ahoga J, et al. Can the performance of pyrethroid-chlorfenapyr nets be reduced when combined with pyrethroid-piperonyl butoxide (PBO) nets? Malar J. 2023;22:214. 37480030 10.1186/s12936-023-04648-6 PMC10362717

[25] Ngufor C, Agbevo A, Fagbohoun J, Fongnikin A, Rowland M. Efficacy of Royal Guard, a new alpha-cypermethrin and pyriproxyfen treated mosquito net, against pyrethroid-resistant malaria vectors. Sci Rep. 2020;10:12227. 32699237 10.1038/s41598-020-69109-5 PMC7376134

[26] WHO. List of WHO prequalified vector control products. Geneva: World Health Organization; 2024.

[27] Black BC, Hollingworth RM, Ahammadsahib KI, Kukel CD, Donovan S. Insecticidal action and mitochondrial uncoupling activity of AC-303,630 and related halogenated pyrroles. Pesticide Biochem Physiol. 1994;50:115–28.

[28] Christophers SR. The development of the egg follicle in anophelines. Paludism. 1911;2:73–88.

[29] WHO. Manual for monitoring insecticide resistance in mosquito vectors and selecting appropriate interventions. Geneva: World Health Organization; 2022.

[30] Myers A, Fagbohoun J, Houetohossou G, Ndombidje B, Govoetchan R, Todjinou D, et al. Identifying suitable methods for evaluating the sterilising effects of pyriproxyfen on adult malaria vectors: a comparison of the oviposition and ovary dissection methods. Malar J. 2024;23:164. 38789998 10.1186/s12936-024-04983-2 PMC11127354

[31] Syme T, N’dombidjé B, Gbegbo M, Todjinou D, Ariori V, De Vos P, et al. PermaNet^®^ dual, a new deltamethrin-chlorfenapyr mixture net, shows improved efficacy against pyrethroid-resistant *Anopheles gambiae sensu lato* in southern Benin. bioRxiv. 2023;8:e1001091. 10.1038/s41598-023-39140-3 PMC10382523 37507423

[32] Yovogan B, Sovi A, Padonou GG, Adoha CJ, Akinro B, Chitou S, et al. Pre-intervention characteristics of the mosquito species in Benin in preparation for a randomized controlled trial assessing the efficacy of dual active-ingredient long-lasting insecticidal nets for controlling insecticide-resistant malaria vectors. PLoS ONE. 2021;16: e0251742. 34014982 10.1371/journal.pone.0251742 PMC8136630

[33] Tchouakui M, Thiomela RF, Nchoutpouen E, Menze BD, Ndo C, Achu D, et al. High efficacy of chlorfenapyr-based net Interceptor^®^ G2 against pyrethroid-resistant malaria vectors from Cameroon. Infect Dis Poverty. 2023;12:81. 37641108 10.1186/s40249-023-01132-w PMC10463949

[34] Zahouli JZB, Edi CAV, Yao LA, Lisro EG, Adou M, Koné I, et al. Small-scale field evaluation of PermaNet(^®^) Dual (a long-lasting net coated with a mixture of chlorfenapyr and deltamethrin) against pyrethroid-resistant *Anopheles gambiae* mosquitoes from Tiassalé, Côte d’Ivoire. Malar J. 2023;22:36. 36726160 10.1186/s12936-023-04455-z PMC9893697

[35] Martin JL, Messenger LA, Rowland M, Mosha FW, Bernard E, Kisamo M, et al. Bio-efficacy of field aged novel class of long-lasting insecticidal nets, against pyrethroid-resistant malaria vectors in Tanzania: a series of experimental hut trials. medRxiv. 2023;391:1577. 10.1371/journal.pgph.0002586 PMC11451999 39365782

[36] Mosha JF, Matowo NS, Kulkarni MA, Messenger LA, Lukole E, Mallya E, et al. Effectiveness of long-lasting insecticidal nets with pyriproxyfen–pyrethroid, chlorfenapyr–pyrethroid, or piperonyl butoxide–pyrethroid versus pyrethroid only against malaria in Tanzania: final-year results of a four-arm, single-blind, cluster-randomised trial. Lancet Infect Dis. 2024;24:87–97. 37776879 10.1016/S1473-3099(23)00420-6

[37] PATH. New Nets Project Interim Results; Output 3: Evidence of effectiveness and cost-effectiveness of dual-AI ITNs created and disseminated. PATH; 2022. Available from: https://www.path.org/our-impact/resources/new-nets-project-interim-results-output-3/.

[38] WHO. Guiding principles for prioritizing malaria interventions in resource constrained country contexts to achieve maximum impact. Geneva: World Health Organization; 2024.

[39] N’Guessan R, Asidi A, Boko P, Odjo A, Akogbeto M, Pigeon O, et al. An experimental hut evaluation of PermaNet® 3.0, a deltamethrin—piperonyl butoxide combination net, against pyrethroid-resistant Anopheles gambiae and Culex quinquefasciatus mosquitoes in southern Benin. Trans R Soc Trop Med Hyg. 2010;104:758–65. 20956008 10.1016/j.trstmh.2010.08.008

[40] Bayili K, N’Do S, Yadav RS, Namountougou M, Ouattara A, Dabiré RK, et al. Experimental hut evaluation of DawaPlus 3.0 LN and DawaPlus 40 LN treated with deltamethrin and PBO against free-flying populations of *Anopheles gambiae s.l.* in Vallée du Kou, Burkina Faso. PLoS ONE. 2019;14: e0226191. 31869350 10.1371/journal.pone.0226191 PMC6927612

[41] Menze BD, Kouamo MF, Wondji MJ, Tchapga W, Tchoupo M, Kusimo MO, et al. An experimental hut evaluation of PBO-based and pyrethroid-only nets against the malaria vector *Anopheles funestus* reveals a loss of bed nets efficacy associated with GSTe2 metabolic resistance. Genes. 2020;11:143. 32013227 10.3390/genes11020143 PMC7073577

[42] Syme T, Gbegbo M, Obuobi D, Fongnikin A, Agbevo A, Todjinou D, et al. Pyrethroid-piperonyl butoxide (PBO) nets reduce the efficacy of indoor residual spraying with pirimiphos-methyl against pyrethroid-resistant malaria vectors. Sci Rep. 2022;12:6857. 35478216 10.1038/s41598-022-10953-y PMC9046380

[43] Dadzie SK, Chabi J, Asafu-Adjaye A, Owusu-Akrofi O, Baffoe-Wilmot A, Malm K, et al. Evaluation of piperonyl butoxide in enhancing the efficacy of pyrethroid insecticides against resistant *Anopheles gambiae* s.l. in Ghana. Malar J. 2017;16:342. 28818077 10.1186/s12936-017-1960-3 PMC5561623

[44] Ogutu N, Agumba S, Moshi V, Ouma C, Ramaita E, Kariuki L, et al. Efficacy of the PermaNet Dual compared to the Interceptor G2 and the PermaNet 3.0 in experimental huts in Siaya County, western Kenya. Res Square. 2024;526:7572. 10.1186/s12936-024-05157-w PMC11531690 39488707

[45] Tungu P, Magesa S, Maxwell C, Malima R, Masue D, Sudi W, et al. Evaluation of PermaNet 3.0 a deltamethrin-PBO combination net against *Anopheles gambiae* and pyrethroid resistant Culex quinquefasciatus mosquitoes: an experimental hut trial in Tanzania. Malar J. 2010;9:21. 20085631 10.1186/1475-2875-9-21 PMC2817703

[46] Sih C, Protopopoff N, Koffi AA, Ahoua Alou LP, Dangbenon E, Messenger LA, et al. Efficacy of chlorfenapyr-pyrethroid and piperonyl butoxide-pyrethroid long-lasting insecticidal nets (LLINs) compared to pyrethroid-only LLINs for malaria control in Côte d’Ivoire: a three group, cluster randomised trial. Trials. 2024;25:151. 38419075 10.1186/s13063-024-07969-2 PMC10900640

[47] Grisales N, Lees RS, Maas J, Morgan JC, Wangrawa DW, Guelbeogo WM, et al. Pyriproxyfen-treated bed nets reduce reproductive fitness and longevity of pyrethroid-resistant *Anopheles gambiae* under laboratory and field conditions. Malar J. 2021;20:273. 34158066 10.1186/s12936-021-03794-z PMC8218427

[48] Ohashi K, Nakada K, Ishiwatari T, Miyaguchi J, Shono Y, Lucas JR, et al. Efficacy of pyriproxyfen-treated nets in sterilizing and shortening the longevity of *Anopheles gambiae* (Diptera: Culicidae). J Med Entomol. 2014;49:1052–8. 10.1603/me12006 23025186

[49] Koama B, Namountougou M, Sanou R, Ndo S, Ouattara A, Dabiré RK, et al. The sterilizing effect of pyriproxyfen on the malaria vector *Anopheles gambiae*: physiological impact on ovaries development. Malar J. 2015;14:101. 25880844 10.1186/s12936-015-0609-3 PMC4355148

[50] Yunta C, Grisales N, Nász S, Hemmings K, Pignatelli P, Voice M, et al. Pyriproxyfen is metabolized by P450s associated with pyrethroid resistance in *An. gambiae*. Insect Biochem Mol Biol. 2016;78:50–7. 27613592 10.1016/j.ibmb.2016.09.001 PMC6399515

[51] Yunta C, Hemmings K, Stevenson B, Koekemoer LL, Matambo T, Pignatelli P, et al. Cross-resistance profiles of malaria mosquito P450s associated with pyrethroid resistance against WHO insecticides. Pesticide Biochem Physiol. 2019;161:61–7. 10.1016/j.pestbp.2019.06.007 31685198

