# Supplementary material for: The experimental hut efficacy of next-generation insecticide-treated nets against pyrethroid-resistant malaria vectors after 12, 24 and 36 months of household use in Benin
Source: Malar J. 2024 Dec 18;23:388. doi: 10.1186/s12936-024-05199-0 (PMC11656845; doi:10.1186/s12936-024-05199-0)
Supplement: Supplementary file 1 — Supplementary Material 1 [file 12936_2024_5199_MOESM1_ESM.docx]

**Table S1** **Experimental hut results of pyrethroid-resistant *Anopheles gambiae* sensu lato entering experimental huts in Covè, Benin.**

| **Net type** | **Untreated net** | **Interceptor** | | | | **PermaNet 3.0** | | | | **Royal Guard** | | | | **Interceptor G2** | | | |
| --- | --- | --- | --- | --- | --- | --- | --- | --- | --- | --- | --- | --- | --- | --- | --- | --- | --- |
| **Age status** | ̶ | **New** | **12 m** | **24 m** | **36 m** | **New** | **12 m** | **24 m** | **36 m** | **New** | **12 m** | **24 m** | **36 m** | **New** | **12 m** | **24 m** | **36 m** |
| ***N* replicate nets** | 24 | 24 | 84 | 54 | 54 | 24 | 84 | 54 | 54 | 24 | 84 | 54 | 54 | 24 | 84 | 54 | 54 |
| ***N* holed nets** | 24 | 24 | 67 | 48 | 50 | 24 | 46 | 47 | 44 | 24 | 66 | 47 | 44 | 24 | 63 | 45 | 45 |
| **% Holed nets** | 100 | 100 | 79.8 | 88.9 | 92.6 | 100 | 54.8 | 87.0 | 81.5 | 100 | 78.6 | 87.0 | 81.5 | 100 | 75.0 | 83.3 | 83.3 |
| **95% confidence intervals** | ̶ | ̶ | 71.2-88.4 | 80.5-97.3 | 85.6-99.6 | ̶ | 44.2-65.4 | 78.0-96.0 | 71.1-91.9 | ̶ | 69.8-87.4 | 78.0-96.0 | 71.1-91.9 | ̶ | 65.7-84.3 | 73.4-93.2 | 73.4-93.2 |
| **Mean proportional hole index** | 138.0 | 138.0 | 110.6 | 457.9 | 497.8 | 138.0 | 86.1 | 320.6 | 384.2 | 138.0 | 246.9 | 528.0 | 418.1 | 138.0 | 139.4 | 819.8 | 598.0 |
| ***N* collected** | 3060 | 1991 | 774 | 797 | 599 | 1708 | 795 | 1127 | 682 | 3340 | 867 | 1120 | 786 | 2123 | 627 | 858 | 728 |
| **Mean collected per night*** | 14.2^ab^ | 9.2^cd^ | 7.2^ef^ | 14.8^ab^ | 11.1^cg^ | 7.9^de^ | 7.4^ef^ | 20.9^h^ | 12.6^ag^ | 15.5^b^ | 8.0^de^ | 20.7^h^ | 14.6^ab^ | 9.8^cd^ | 5.8^f^ | 15.9^b^ | 13.5^abg^ |
| **% Deterrence** | ̶ | 35.2 | 49.3 | -0.7 | -4.2 | 44.4 | 47.9 | -3.5 | -47.2 | -9.2 | 43.7 | -13.4 | -45.8 | 31.0 | 59.2 | 18.3 | -12.0 |
| ***N* exiting** | 1195 | 1115 | 532 | 549 | 395 | 1304 | 612 | 825 | 484 | 2378 | 676 | 813 | 603 | 1329 | 471 | 598 | 514 |
| **% Exophily*** | 39.1^a^ | 56.0^b^ | 68.7^cdef^ | 68.9^cde^ | 65.9^cg^ | 76.3^defh^ | 77.0^i^ | 73.2^fhi^ | 71.0^cdefh^ | 71.2^c^ | 78.0^hi^ | 72.6^dfhi^ | 76.7^cde^ | 62.6^g^ | 75.1^hi^ | 69.7^cde^ | 70.6^ce^ |
| **95% confidence intervals** | 37.4-40.8 | 53.8-58.2 | 65.4-72.0 | 65.7-72.1 | 62.1-69.7 | 74.3-78.3 | 74.1-79.9 | 70.6-75.8 | 67.6-74.4 | 69.7-72.7 | 75.2-80.8 | 70.0-75.2 | 73.7-79.7 | 60.5-64.7 | 71.7-78.5 | 66.6-72.8 | 67.3-73.9 |
| ***N* inside net** | 1251 | 465 | 24 | 5 | 1 | 163 | 11 | 1 | 4 | 363 | 3 | 2 | 3 | 379 | 1 | 1 | 5 |
| **% Inside net*** | 40.9^a^ | 23.4^b^ | 3.1^c^ | 0.6^def^ | 0.2^def^ | 9.5^g^ | 1.4^d^ | 0.1^e^ | 0.6^def^ | 10.9^g^ | 0.3^ef^ | 0.2^ef^ | 0.4^def^ | 17.9^h^ | 0.2^ef^ | 0.1^ef^ | 0.7^df^ |
| **95% confidence intervals** | 39.2-42.6 | 21.5-25.3 | 1.9-4.3 | 0.1-1.1 | 0.0-0.6 | 8.1-10.9 | 0.6-2.2 | 0.0-0.3 | 0.0-1.2 | 9.8-12.0 | 0.0-0.7 | 0.0-0.5 | 0.0-0.8 | 16.3-19.5 | 0.0-0.5 | 0.0-0.3 | 0.1-1.3 |
| ***N* unfed live** | 1114 | 860 | 449 | 515 | 377 | 816 | 462 | 726 | 408 | 1814 | 579 | 798 | 479 | 446 | 232 | 426 | 340 |
| ***N* unfed dead** | 24 | 226 | 114 | 126 | 51 | 548 | 151 | 161 | 113 | 911 | 172 | 180 | 107 | 847 | 208 | 248 | 137 |
| ***N* blood-fed live** | 1914 | 837 | 195 | 148 | 156 | 265 | 171 | 222 | 149 | 430 | 105 | 128 | 193 | 450 | 167 | 163 | 241 |
| ***N* blood-fed dead** | 8 | 68 | 16 | 8 | 15 | 79 | 11 | 18 | 12 | 185 | 11 | 14 | 7 | 380 | 20 | 21 | 10 |
| ***N* blood-fed** | 1922 | 905 | 211 | 156 | 171 | 344 | 182 | 240 | 161 | 615 | 116 | 142 | 200 | 830 | 187 | 184 | 251 |
| **% Blood-feeding*** | 62.8^a^ | 45.5^b^ | 27.3^cde^ | 19.6^c^ | 28.5^df^ | 20.1^de^ | 22.9^ce^ | 21.3^de^ | 23.6^def^ | 18.4^cde^ | 13.4^g^ | 12.7^g^ | 25.4^def^ | 39.1^h^ | 29.8^de^ | 21.4^def^ | 34.5^f^ |
| **95% confidence intervals** | 61.1-64.5 | 43.3-47.7 | 24.2-30.4 | 16.8-22.4 | 24.9-32.1 | 18.2-22.0 | 20.0-25.8 | 18.9-23.7 | 20.4-26.8 | 17.1-19.7 | 11.1-15.7 | 10.7-14.7 | 22.4-28.4 | 37.0-41.2 | 26.2-33.4 | 18.7-24.1 | 31.0-38.0 |
| **% Blood-feeding inhibition** | ̶ | 27.5 | 56.5 | 68.8 | 54.6 | 68.0 | 63.5 | 66.1 | 62.4 | 70.7 | 78.7 | 79.8 | 59.6 | 37.7 | 52.5 | 65.9 | 45.1 |
| **% Personal protection** | ̶ | 52.9 | 89.0 | 91.9 | 91.1 | 82.1 | 90.5 | 87.5 | 91.6 | 68.0 | 94.0 | 92.6 | 89.6 | 56.8 | 90.3 | 90.4 | 86.9 |
| ***N* immediate mortality** | 18 | 103 | 59 | 23 | 21 | 360 | 67 | 59 | 39 | 565 | 78 | 47 | 34 | 720 | 143 | 159 | 67 |
| **% Immediate mortality** | 0.6 | 5.2 | 7.6 | 2.9 | 3.5 | 21.1 | 8.4 | 5.2 | 5.7 | 16.9 | 9.0 | 4.2 | 4.3 | 33.9 | 22.8 | 18.5 | 9.2 |
| **95% confidence intervals** | 0.3–0.9 | 4.2–6.2 | 5.7–9.5 | 1.7–4.1 | 2.0–5.0 | 19.2–23.0 | 6.5–10.3 | 3.9–6.5 | 4.0–7.4 | 15.6–18.2 | 7.1–10.9 | 3.0–5.4 | 2.9–5.7 | 31.9–35.9 | 19.5–26.1 | 15.9–21.1 | 7.1–11.3 |
| ***N* 24 h mortality** | 28 | 246 | 112 | 103 | 52 | 579 | 149 | 156 | 84 | 993 | 165 | 157 | 84 | 1113 | 207 | 255 | 130 |
| **% 24 h mortality** | 0.9 | 12.4 | 14.5 | 12.9 | 8.7 | 33.9 | 18.7 | 13.8 | 12.3 | 29.7 | 19.0 | 14.0 | 10.7 | 52.4 | 33.0 | 29.7 | 17.9 |
| **95% confidence intervals** | 0.6-1.2 | 11.0-13.8 | 12.0-17.0 | 10.6-15.2 | 6.4-11.0 | 31.7-36.1 | 16.0-21.4 | 11.8-15.8 | 9.8-14.8 | 28.2-31.2 | 16.4-21.6 | 12.0-16.0 | 8.5-12.9 | 50.3-54.5 | 29.3-36.7 | 26.6-32.8 | 15.1-20.7 |
| ***N* 48 h mortality** | 30 | 275 | 120 | 127 | 59 | 609 | 154 | 163 | 104 | 1042 | 178 | 175 | 92 | 1176 | 218 | 261 | 142 |
| **% 48 h mortality** | 1.0 | 13.8 | 15.5 | 15.9 | 9.8 | 35.7 | 19.4 | 14.5 | 15.2 | 31.2 | 20.5 | 15.6 | 11.7 | 55.4 | 34.8 | 30.4 | 19.5 |
| **95% confidence intervals** | 0.6-1.4 | 12.3-15.3 | 13.0-18.0 | 13.4-18.4 | 7.4-12.2 | 33.4-38.0 | 16.7-22.1 | 12.4-16.6 | 12.5-17.9 | 29.6-32.8 | 17.8-23.2 | 13.5-17.7 | 9.5-13.9 | 53.3-57.5 | 31.1-38.5 | 27.3-33.5 | 16.6-22.4 |
| ***N* 72 h mortality** | 32 | 294 | 130 | 134 | 66 | 627 | 162 | 179 | 125 | 1096 | 183 | 194 | 114 | 1227 | 228 | 269 | 147 |
| **% 72 h mortality*** | 1.0^a^ | 14.8^bc^ | 16.8^de^ | 16.8^de^ | 11.0^b^ | 36.7^f^ | 20.4^d^ | 15.9^bc^ | 18.3^ce^ | 32.8^g^ | 21.1^de^ | 17.3^de^ | 14.5^bc^ | 57.8^h^ | 36.4^f^ | 31.4^f^ | 20.2^d^ |
| **95% confidence intervals** | 0.6-1.4 | 13.2-16.4 | 14.2-19.4 | 14.2-19.4 | 8.5-13.5 | 34.4-39.0 | 17.6-23.2 | 13.8-18.0 | 15.4-21.2 | 31.2-34.4 | 18.4-23.8 | 15.1-19.5 | 12.0-17.0 | 55.7-59.9 | 32.6-40.2 | 28.3-34.5 | 17.3-23.1 |
| **% Overall killing effect** | ̶ | 8.6 | 3.2 | 3.3 | 1.1 | 19.4 | 4.2 | 4.8 | 3 | 34.8 | 4.9 | 5.3 | 2.7 | 39.1 | 6.4 | 7.7 | 3.8 |
| ***N* dissected** | 1572 | 795 | 195 | 135 | 155 | 265 | 167 | 161 | 148 | 384 | 103 | 112 | 192 | 448 | 157 | 155 | 227 |
| ***N* fertile** | 1449 | 704 | 149 | 121 | 150 | 225 | 135 | 145 | 137 | 87 | 66 | 92 | 132 | 378 | 108 | 131 | 210 |
| **% Fertile*** | 92.2^a^ | 88.6^b^ | 76.4^bcd^ | 89.6^ab^ | 96.8^abc^ | 84.9^bc^ | 80.8^b^ | 90.1^de^ | 92.6^abc^ | 22.7^f^ | 64.1^de^ | 82.1^bcde^ | 68.8^e^ | 84.4^bc^ | 68.8^cd^ | 84.5^bcd^ | 92.5^abc^ |
| **95% confidence intervals** | 90.9-93.5 | 86.4-90.8 | 70.4-82.4 | 84.5-94.7 | 94.0-99.6 | 80.6-89.2 | 74.8-86.8 | 85.5-94.7 | 88.4-96.8 | 18.5-26.9 | 54.8-73.4 | 75.0-89.2 | 62.2-75.4 | 81.0-87.8 | 61.6-76.0 | 78.8-90.2 | 89.1-95.9 |
| **% Reduction in fertility** | ̶ | 3.9 | 17.1 | 2.8 | -5.0 | 7.9 | 12.4 | 2.3 | -0.4 | 75.4 | 30.5 | 11.0 | 25.4 | 8.5 | 25.4 | 8.4 | -0.3 |

*According to regression analysis, values in the same row sharing a common superscript letter are not significantly different at the 5% level (p>0.05). Data with untreated control and new nets is pooled across different trials to provide a single efficacy estimate.
