# Supplementary material for: The experimental hut efficacy of next-generation insecticide-treated nets against pyrethroid-resistant malaria vectors after 12, 24 and 36 months of household use in Benin
Source: Malar J. 2024 Dec 18;23:388. doi: 10.1186/s12936-024-05199-0 (PMC11656845; doi:10.1186/s12936-024-05199-0)
Supplement: Supplementary file 2 — Supplementary Material 2 [file 12936_2024_5199_MOESM2_ESM.docx]

**Table S2 Chemical analysis results showing mean chemical content of nets at each annual time point.**

| **Time point** | **Net type** | **Interceptor** | **PermaNet 3.0** | | | **Royal Guard** | | **Interceptor G2** | |
| --- | --- | --- | --- | --- | --- | --- | --- | --- | --- |
|  | **Active ingredient (AI)** | **Alpha-cypermethrin** | **Deltamethrin (sides)** | **Deltamethrin (roof)** | **Piperonyl butoxide (roof)** | **Alpha-cypermethrin** | **Pyriproxyfen** | **Alpha-cypermethrin** | **Chlorfenapyr** |
| **New** | **Mean AI content (g/kg)** | 6.3 | 1.9 | 4.0 | 20.8 | 5.8 | 6.3 | 2.6 | 5.0 |
|  | **95% CIs** | 6.0-6.5 | 1.8-2.1 | 3.9-4.1 | 20.0-21.6 | 5.7-5.9 | 6.3-6.4 | 2.5-2.7 | 4.8-5.1 |
|  | **% RSD** | 10.9 | 0.7 | 0.3 | 0.4 | 4.4 | 3 | 8.7 | 8.7 |
| **12 months** | **Mean AI content (g/kg)** | 1.9 | ̶ | ̶ | ̶ | 4.4 | 3.0 | 1.6 | 2.0 |
|  | **95% CIs** | 1.6-2.3 | ̶ | ̶ | ̶ | 4.1-4.7 | 2.6-3.4 | 1.4-1.8 | 1.5-2.5 |
|  | **% RSD** | 44.4 | ̶ | ̶ | ̶ | 18.5 | 37.9 | 34.3 | 71 |
|  | **% AI retention** | 30.8 | ̶ | ̶ | ̶ | 75.8 | 47.0 | 62.3 | 40.2 |
| **24 months** | **Mean AI content (g/kg)** | 1.6 | ̶ | ̶ | ̶ | 3.6 | 1.8 | 1.2 | 1.2 |
|  | **95% CIs** | 1.1-2.2 | ̶ | ̶ | ̶ | 3.2-3.9 | 1.5-2.2 | 1.0-1.4 | 0.8-1.7 |
|  | **% RSD** | 94.3 | ̶ | ̶ | ̶ | 24.7 | 44.4 | 47.8 | 101.4 |
|  | **% AI retention** | 26.0 | ̶ | ̶ | ̶ | 61.5 | 29.1 | 46.9 | 24.3 |
| **36 months** | **Mean AI content (g/kg)** | 0.9 | 0.3 | 2.7 | 5.7 | 3.1 | 1.7 | 1.0 | 0.7 |
|  | **95% CIs** | 0.6-1.2 | 0.1-0.4 | 2.6-2.8 | 4.7-6.7 | 2.7-3.5 | 1.3-2.1 | 0.8-1.2 | 0.4-1.1 |
|  | **% RSD** | 90.6 | 150.1 | 12.7 | 45.9 | 33.7 | 66.3 | 60 | 125.3 |
|  | **% AI retention** | 14.9 | 14.0 | 68.4 | 27.4 | 53.2 | 26.4 | 37.3 | 14.7 |

CI=Confidence interval, RSD=Relative standard deviation. Proportional active ingredient retention is calculated relative to new nets at each time point. Chemical analysis was not performed with PermaNet® 3.0 at 12 and 24 months due to funding constraints.
